# Knowledge and Practice of Incremental Hemodialysis: A Survey of Canadian Nephrologists

**DOI:** 10.1177/20543581211065255

**Published:** 2021-12-17

**Authors:** Anita Dahiya, Aminu Bello, Stephanie Thompson, Kara Schick-Makaroff, Neesh Pannu

**Affiliations:** 1 Department of Medicine, University of Alberta, Edmonton, Canada; 2 Department of Medicine, Division of Nephrology, University of Alberta, Edmonton, Canada; 3 Faculty of Nursing, University of Alberta, Edmonton, Canada

**Keywords:** incremental hemodialysis, end-stage kidney disease, hemodialysis prescription, survey

## Abstract

**Background:**

Incremental hemodialysis, a strategy to individualize dialysis prescription based on residual kidney function, may be associated with enhanced quality of life and decreased health care costs compared with conventional hemodialysis.

**Objective:**

We surveyed practicing Canadian nephrologists to assess knowledge, perceptions, and practice pattern on the use of incremental hemodialysis.

**Design/Setting:**

We distributed a cross-sectional, web-based survey. We asked about incremental hemodialysis prescribing practices, including frequency of prescription, clinical factors used to determine suitability for treatment, and barriers to implementation. The survey was conducted from September 21 to October 30, 2020.

**Participants:**

We distributed the survey to practicing Canadian nephrologists identified from a private membership list of the Canadian Society of Nephrology (CSN), as well as to nephrologists named on a publicly available national list of practicing Canadian nephrologists created from provincial College of Physician registries. These were samples of convenience.

**Methods:**

We conducted descriptive analysis of categorical data including frequencies for nominal variables and measures of central tendency (mean) and dispersion (standard deviation) for ordinal variables. We used chi-square analysis to identify association between participant and practice characteristics and their opinions and attitudes toward incremental dialysis. We used simple thematic analysis on free-text responses on questions regarding the prescription of incremental hemodialysis, focusing on age and baseline management of cardiac and noncardiac comorbidities.

**Results:**

The response rate was 35% (243/691). Most (138/211, 65%) of the participants prescribed incremental hemodialysis using an individualized approach at the nephrologist’s discretion. Most participants (200/203, 98%) did not report any policy for implementation. Residual urine output was identified as the most important factor for eligibility (112/172, 65%), followed by electrolyte stability (76/172, 44%) and patient goals of care (69/117, 40%). Most participants agreed that dialysis prescriptions should take residual kidney function into consideration; however, 74% of the participants disagreed with a statement that there was strong evidence supporting incremental hemodialysis. Barriers identified included patient safety, patient acceptance of dose escalation, and logistics of scheduling. Despite these barriers, 82% of participants felt that that incremental hemodialysis is feasible with their current resources and 78% agreed that with specific criteria, it is a safe option.

**Limitations:**

The generalizability of our study is limited by its response rate of 35%; however, this is comparable with typical response rates seen in electronic surveys. Most participants practice in an academic setting, which may have introduced bias to the results.

**Conclusions:**

Despite the perception of limited evidence and a lack of guidance on implementation, incremental hemodialysis is frequently practiced by Canadian nephrologists. Barriers to implementation were identified, highlighting the need for research to guide practice.

## Abrégé

**Contexte:**

L’hémodialyse incrémentale est une stratégie tenant compte de la fonction rénale résiduelle pour individualiser la prescription de dialyse. Comparée à l’hémodialyse conventionnelle, cette modalité pourrait être associée à une amélioration de la qualité de vie des patients et à une réduction des coûts de santé.

**Objectif:**

Nous avons interrogé des néphrologues canadiens en exercice afin d’évaluer leurs connaissances, leurs perceptions et leurs habitudes de pratique relativement à l’hémodialyse incrémentale.

**Conception de l’étude:**

Une enquête transversale en ligne a été distribuée. Les questions portaient sur les pratiques de prescription de l’hémodialyse incrémentale, notamment sur la fréquence de prescription, les facteurs cliniques utilisés pour déterminer l’adéquation du traitement, et les obstacles à la mise en œuvre. Le sondage a été réalisé entre le 21 septembre et le 30 octobre 2020.

**Participants:**

Nous avons distribué le sondage aux néphrologues canadiens en exercice figurant sur une liste privée des membres de la Société canadienne de néphrologie (SCN), ainsi qu’aux néphrologues figurant sur une liste nationale publique des néphrologues canadiens en exercice, créée à partir des registres des Collèges des médecins provinciaux. Il s’agit d’échantillons de commodité.

**Méthodologie:**

Nous avons procédé à une analyse descriptive des données catégoriques, notamment des fréquences pour les variables nominales et des mesures de tendance centrale (moyenne) et de dispersion (écart-type) pour les variables ordinales. Nous avons utilisé l’analyse du chi-carré pour établir l’association des caractéristiques des répondants et de leurs pratiques avec leurs opinions et comportements à l’égard de la dialyse incrémentale. Nous avons procédé à une analyse thématique simple des réponses en texte libre aux questions portant sur la prescription de l’hémodialyse incrémentale, en se concentrant sur l’âge et la prise en charge initiale des comorbidités cardiaques et non cardiaques.

**Résultats:**

Le taux de réponse était de 35 % (243/691). Une majorité de répondants (138/211 [65 %]) prescrivaient l’hémodialyse incrémentale selon une approche individualisée à la discrétion du néphrologue. La quasi-totalité des répondants (200/203 [98 %]) n’a pas fait état d’une politique de mise en œuvre. La diurèse résiduelle a été désignée comme le principal facteur d’admissibilité (112/172 [65 %]), suivie de la stabilité électrolytique (76/172 [44 %]) et des objectifs de soins du patient (69/117 [40 %]). La plupart des répondants ont convenu que la prescription de dialyse devrait tenir compte de la fonction rénale résiduelle; 74 % d’entre eux se sont toutefois dits en désaccord avec une affirmation selon laquelle il existe de solides preuves appuyant l’hémodialyse incrémentale. La sécurité du patient, l’acceptation par le patient d’une augmentation de la dose et la logistique de la planification figurent parmi les obstacles à la mise en œuvre mentionnés par les répondants. Malgré ces obstacles, 82 % des répondants étaient d’avis que l’hémodialyse incrémentale est possible avec les ressources actuelles, et 78 % étaient d’accord pour dire qu’avec des critères précis, cette modalité est une option sûre.

**Limites:**

La généralisabilité de notre étude est limitée par son faible taux de réponse (35 %), bien que celui-ci soit comparable à ceux qu’on observe généralement pour les enquêtes électroniques. La plupart des répondants exerçaient dans un cadre académique, ce qui peut avoir introduit un biais dans les résultats.

**Conclusion:**

L’hémodialyse incrémentale est fréquemment prescrite par les néphrologues canadiens, malgré une perception de preuves limitées et un manque d’orientation pour sa mise en œuvre. Les répondants ont mentionné quelques obstacles à sa mise en œuvre, soulignant ainsi la nécessité de mener des recherches afin de mieux orienter la pratique.

## Introduction

The number of patients requiring kidney replacement therapy in Canada is increasing annually due to shifts in population demographics and better survival of patients with end-stage kidney disease.^1-3^ Despite the increase in uptake of home-based dialysis therapies, more than 80% of patients with end-stage kidney disease in Canada are treated with facility-based hemodialysis.^2^

The transition to dialysis is a challenging experience for most patients and is associated with a marked increase in hospitalization, morbidity, and mortality in the first 3 months.^4^ The current dialysis prescription of establishing a Kt/V_urea_ of 1.2 with thrice-weekly dialysis was established by the Kidney Disease Outcomes Quality Initiative (KDOQI) based on the landmark National Cooperative Dialysis Study (NCDS) and Hemodialysis Study (HEMO) trials.^5,6^ Unfortunately, conventional hemodialysis prescriptions do not account for residual kidney function (RKF), which has previously been described as an important determinant of dialysis requirements and has been associated with improved cardiovascular outcomes, nutrition, phosphorous control, and middle molecule clearance.^7,8^ Furthermore, conventional hemodialysis is associated with repeated ischemic insults, which can contribute to rapid decline in RKF.^9^

At present, most patients initiating hemodialysis, regardless of age, sex, comorbidity, weight, and RKF are given the same treatment prescription. Incremental hemodialysis has been proposed as a “gentler” approach to hemodialysis initiation, where the frequency and duration of dialysis sessions are individualized to account for RKF, increasing treatment delivery frequency and/or duration as kidney function decreases.^10^ Regular monitoring of patients for uremic symptoms, volume status, and RKF through 24-h urine collection is performed. The 2015 update of the KDOQI clinical practice guidelines and the European Best Practices guidelines endorse this approach by suggesting RKF should be considered in the prescription of hemodialysis with modification of minimal adequacy targets; however, this has not been widely translated into clinical practice.^11,12^

An incremental approach to hemodialysis initiation may facilitate delivery of patient centered care while reducing the per patient cost of care delivery and increasing treatment capacity.^13^ Anecdotally, it is known that there is use of incremental hemodialysis in Canada; however, the extent of use and current practice is unclear. We surveyed practicing Canadian nephrologists to assess knowledge, perceptions, and practice pattern on the use of incremental hemodialysis.

## Methods

### Design and Participants

A cross-sectional survey of nephrologists practicing in Canada was implemented using an online survey tool. The convenience sampling frame was practicing Canadian nephrologists, who were identified from a private membership list of the Canadian Society of Nephrology (CSN) as well as a publicly available national list of practicing Canadian nephrologists created from provincial College of Physician registries. Duplicates were removed from the combined list at the time of survey distribution so that nephrologists would be contacted once, yielding a final list of 691 practicing nephrologists in Canada with a publicly available email address.

### Development and Validation of Survey Instrument

The first and last authors (A.D. and N.P.) developed a survey to assess attitudes toward and current practices of incremental hemodialysis. For the purposes of the survey, incremental hemodialysis was defined as prescription of facility-based hemodialysis in new patients (within 12 months) where hemodialysis is titrated based on RKF. This definition refers to hemodialysis started at less than 3 times per week and/or less than 4 h per session with the intention to titrate the treatment as needed. Items were generated through a review of the current literature and a focus group with 3 nephrologists. The pilot survey was peer reviewed for content validity and comprehensiveness. Pilot participants included 5 academic nephrologists with expertise in survey design, as well as an implementation scientist. The survey was revised based on their recommendations and subsequently formatted for online administration. The final questionnaire included 22 questions eliciting knowledge of incremental hemodialysis, opinions regarding various dialysis prescription factors, and perception of feasibility of incremental hemodialysis along with items eliciting demographic and practice characteristics of each participant (Supplementary Appendix 1). The demographic data included location of training, years in practice, dialysis provision model, practice style (academic vs private practice), and reimbursement structure (alternative relationship plan vs fee for service). Dialysis provision models included longitudinal care, in which nephrologists follow select patients throughout their dialysis care, shift-based, in which nephrologists follow patients during a designated dialysis shift, or a combination of the above models.

The final survey, including a combination of multiple-choice and free-text questions, was uploaded to the Select Survey platform (https://survey.albertahealthservices.ca), an online platform that provides anonymity for its users and is copyrighted by Alberta Health Services, the provincial health authority for the province of Alberta, Canada. The survey was only distributed in English.

### Survey Administration

The CSN emailed the survey web link to all identified nephrologists as described above. The survey was delivered over a period of 6 weeks (September 21, 2020, and October 30, 2020). To optimize participation, reminders were sent weekly until the end of the survey period. Participants were informed that the confidentiality of the data would be protected and only aggregate results would be disseminated.

### Data Analysis

The survey results were stored anonymously on the Select Survey platform prior to analysis. The data were subsequently exported to the Excel software (Microsoft Corp, Redmond, WA) for descriptive analysis of the categorical data. This analysis included frequencies for nominal variables and measures of central tendency (mean) and dispersion (standard deviation) for ordinal variables. Chi-square analysis was performed to identify association between participant and practice characteristics and their opinions and attitudes toward incremental dialysis. The level of statistical significance was set at .05.

Simple thematic analysis was used on free-text responses on questions regarding the prescription of incremental hemodialysis, focusing on age and baseline management of cardiac and noncardiac comorbidities.^14^ Participants were also given the opportunity to provide free-text responses on perceived barriers to incremental hemodialysis. On the first level, the responses were read and transcribed independently by the first and last authors of the article (A.D. and N.P.). This was followed by a collaborative effort to determine overarching codes on incremental hemodialysis prescribing practices and perceived barriers to incremental hemodialysis. Finally, the identified themes were reviewed before being defined and written up. This study was approved by the Research Ethics Board at the University of Alberta (Pro00098076). The survey design and reporting was validated using the Checklist for Reporting Results of Internet E-Surveys.^15^

Of the 691 nephrologists that were contacted, 243 responses were received (response rate of 35%). One hundred and sixty-one (66%) responses were 100% complete and 82 (34%) were at least 50% complete. Of these, 6 participants indicated that they did not provide dialysis treatment and were excluded from subsequent analysis; therefore, the total number of participants used in the final analysis was 237. Partially complete surveys were analyzed for the questions answered completely; if a question was not answered fully, it was excluded from analysis of that question.

## Results

### Demographic Information, Including Dialysis Practice, of Surveyed Participants

Characteristics of the participants are provided in Table 1. Most of the participants completed their nephrology training in Canada (92%), with 69% in practice for at least 10 years. Participants commonly had an academic practice (62%) and were reimbursed for dialysis using a fee for service model (69%). Seventy-seven percent of all participants spend more than 50% of their time on clinical work. Forty-four percent of participants used a longitudinal/continuous care model and 44% used a shift-based model of dialysis coverage. Participants providing longitudinal/continuous dialysis care more frequently provided dialysis for greater than 6 months of the year (21%) compared with those that provide shift-based care (8%). In both provision models, patients were most often seen weekly.

**Table 1.** Demographic Information of Survey Participants, Including Dialysis Practice.

Variable, No. (%) unless otherwise specified					*n*	%
Location of nephrology training *n* = 243						
Canada					223	92
Outside of Canada					20	8
Years in practice *n* = 243						
<5 y					49	20
5-9 y					28	12
10-19 y					92	38
>20 y					74	31
Type of practice *n* = 212						
Academic					132	62
Private practice					37	18
Mixed					43	20
Reimbursement structure *n* = 210						
ARP (fixed payment/ARP)					52	25
FFS					146	69
Combination FFS and ARP					4	2
Prefer not to disclose					8	4
Proportion of time spent on clinical work (based on type of practice) *n* = 213						
	Academic		Private		Mixed	
	*n*	%	*n*	%	*n*	%
<30%	1	1	2	1	20	9
30-50%	23	11	0	0	2	1
51-75%	48	23	3	1	7	3
>75%	41	19	34	16	32	15
Provision of dialysis *n* = 243						
Yes					237	97
No					6	3
Dialysis provision model *n* = 229						
Longitudinal/continuous					100	44
Shifts					102	44
Combination					27	12
Frequency of HD coverage (based on the provision model) *n* = 184						
	Longitudinal/continuous		Shifts		Mixed	
	*n*	%	*n*	%	*n*	%
< 3 mo/y	12	7	44	24	6	3
3-6 mo/y	21	11	34	18	4	2
>6 mo/y	38	21	15	8	10	5
Frequency with which patients are seen (based on provision model) *n* = 206						
	Longitudinal/continuous		Shifts		Mixed	
	*n*	%	*n*	%	*n*	%
Thrice weekly	5	2	24	12	5	2
Twice weekly	11	5	8	4	1	1
Weekly	34	16	44	21	12	6
Bi-weekly	20	10	7	3	0	0
Monthly	13	6	8	4	2	1
Every 6 wk	1	1	2	1	1	1
Every 2 mo	2	1	0	0	1	1
Every 3 mo	1	1	2	1	2	1

*Note.* ARP = alternate relationship plan; FFS = fee for service; HD = hemodialysis.

Sixty percent of participants worked in centers where there are more than 50 patients receiving facility-based hemodialysis (see Table 2). Sixty-five percent of participants reported prescribing incremental hemodialysis, with most of the prescriptions individualized. Finally, 98% of participants did not have center-specific criteria available for incremental hemodialysis. There was no significant association between prescription of incremental hemodialysis and participant or practice characteristics (results not shown).

**Table 2.** Surveyed Participants Current Incremental Dialysis Practice.

Variable, No. (%) unless otherwise specified	*n*	%
Number of in-center HD patients *n* = 205		
0	38	2
<10	38	6
10-50	37	29
51-100	42	28
>100	45	32
Unsure	5	4
Provision of incremental dialysis *n* = 211		
Yes	138	65
No	73	35
Current model of incremental dialysis *n* = 111		
Individualized	66	59
Start with once weekly	4	4
Start with twice weekly	32	29
Decreased hours 3 times per week	9	8
Number of new HD starts on incremental dialysis *n* = 202		
<30%	152	75
30-50%	11	5
51-75%	5	2
>75%	9	5
Unsure	25	12
Center-specific criteria available for incremental dialysis *n* = 203		
Yes	3	2
No	200	98

*Note.* HD = hemodialysis.

### Determination of Important Patient and Clinical Factors in Prescribing Incremental Hemodialysis

**Figure 1.**
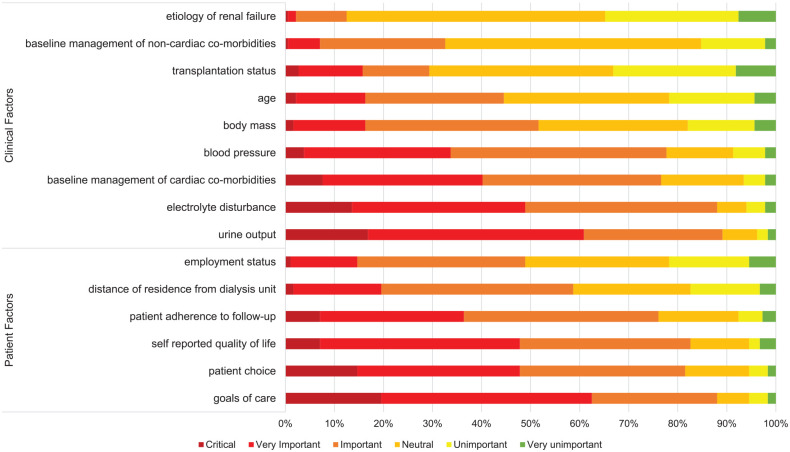
Importance of certain patient and clinical factors in prescribing incremental hemodialysis. *Note.* Responses are represented as a percentage of a total *n* = 184.

Participants were asked to rank a predefined list of patient factors (eg, goals of care), and clinical factors (eg, urine output), that they considered in the decision to provide incremental hemodialysis (see Figure 1). Urine output was the most important factor in the decision to prescribe incremental hemodialysis (65%), followed by electrolyte homeostasis and goals of care (44% and 40%, respectively). Forty-five percent of participants ranked “age” from important to critical. Participants were asked to provide additional details in a free-text response if they ranked “age” as important (see Table 3). Participants were more likely to consider incremental hemodialysis in the elderly population largely due to concerns regarding quality of life. In contrast, participants identified concern of patient acceptance of dose escalation and desire to optimize patients for possible transplantation when considering younger patients for incremental hemodialysis. Poor management of cardiac and volume-related comorbidities was identified as a possible deterrent to initiation of incremental hemodialysis. There was no significant association between ranking of patient and clinical factors in incremental hemodialysis prescriptions and participant or practice characteristics (results not shown).

**Table 3.** Simple Thematic Analysis of Free-Text Responses.

Themes: Opinion on factors which should be considered in prescribing incremental hemodialysis^a^
Age (8/56 quotes displayed)
Older patients are less likely to tolerate conventional HD and focus of care should be centered on quality of life, depending on goals of care
*“More likely in elderly patients especially those with a greater focus on quality of life rather than other parameters”*
*“age is often related to Goal of Care. patient priorities are different in the very elderly often older people may not require as much dialysis and often find starting 3 x week daunting and not understandable, from no dialysis to 3 weekly, so I have found that the gradual approach is more understandable/ acceptable”*
*“Overall the benefit of dialysis in certain age groups is questionable. Generally, in patients > 75, we can not improve quantity of life. The last variable is quality of life. Studies suggest that in frail and institutionalized patients that quality of life decreases with dialysis. However, we are left treating the person in front of us. Therefore, I will trial 2times per week dialysis in some elderly patients to see how the feel about it and if it improves their quality of life/symptoms.”*
*“Age in so far as older patients have potentially greater frailty, Comorbidities, are more likely to have advance care plans and goals of care discussions documented to direct treatment.”*
*“If patient elderly with residual renal function AND has smaller body size/borderline QOL, I would think it reasonable to try incremental hemodialysis if could reasonably control K, volume, etc.”*
Younger patients can tolerate more aggressive dialysis and if a transplant candidate, are less likely to do incremental dialysis to optimize health status
*“May be more aggressive in younger patients”*
*“Younger patients that want to maximize their health status before transplant would be less likely to opt for incremental dialysis and more likely to opt for intensive home hemo.”*
*“Younger: avoid decrease in health changes that may interfere with transplant.”*
Baseline management of cardiac and noncardiac comorbidities (2/10 quotes displayed)
Patients with poor control of cardiovascular comorbidities, particularly volume control, are less likely to be prescribed incremental hemodialysis
*“Volume status and disease related to poor volume control (CHF, ESLD) less likely to prescribe incremental dialysis”*
*“Hypertension, CHF (with or without ventricular failure), angina, exertional dyspnea. If they have poor control of any of these I will lean towards conventional, non-incremental orders. If all are well controlled and the patient is on incremental HD, I emphasize to the patient that if their actions and their dialysis cannot control these risks for premature death/hospitalization then we will need to increase to 3x/week 4h runs.”*
Theme: Perceived barriers to incremental hemodialysis (18/49 quotes displayed)^a^
There is limited supporting scientific evidence in the form of randomized control trials, which is a limitation in the support of incremental hemodialysis
*“Difficult to reconcile the benefits of intensive dialysis (for which there is evidence) and late start vs early (for which there is evidence) with incremental dialysis which would, in some ways, suggest the opposite is reasonable. How do we determine when to start incremental HD?—at what eGFR? with what symptoms? with what parameters do we judge when to escalate therapy? I also fear that this will lead to US-style therapy plans where we will have to negotiate with patients ever increment of 15 minutes. Does incremental HD end at 4 hour treatments? It is a very reasonable hypothesis that benefits will be derived from treatments >4 hours. There are serious questions about the floor and ceiling prescriptions. Lots of questions.”*
*“I think incremental dialysis trials should be done in the context of a good clinical trial to provide use with more guidance on risks and benefits to patients.”*
*“Although my bias is against incremental dialysis (I think many of the issues addressed can be better served by home independent or assisted dialysis), I am willing to accept there may be equipoise and would certainly agree to participate in a trial. I would not at present support a programmatic shift to incremental dialysis without further RCT evidence”*
*“I think the biggest potential problem with incremental dialysis is that it is proposed for people who don’t meet criteria for initiation of HD according to the control group in the IDEAL trial. When we do that we are incurring societal costs and not following evidence. . .”*
*“Lack of evidence/standardization is an issue for program development and consideration of this strategy for younger patients. Structured for 3x/ week dialysis, so twice weekly dialysis leaves a run unfilled in community unit. Difficult to utilize these spots, which causes issues.”*
Centers are often over capacity and there is not enough staff available to accommodate variable hemodialysis schedules
*“Most units are at or over 100% capacity. I’m concerned about the ability to increase frequency when indicated.”*
*“Ongoing capacity constraints create major scheduling challenges to facilitate individual incremental HD prescriptions particularly twice weekly to balance spacing between treatments”*
*“Lack of staff availability and little nursing support”*
*“no spots to start new patients—although some flexibility if only 2/week”*
*“Not many spots, so if many on random 2 times a week schedule, would be a scheduling nightmare.”*
*“No flexibility in scheduling. Too many patients. Potentially chaotic for staff”*
There needs to cooperation from both the staff and patients for incremental hemodialysis to be feasible
*“Patients will become entitled to their less-than-3-times-week or less-than-4-hours schedules and will not accept to modify the prescription. There would be a lot of variability of practice in our group with this approach and a lack of consistency in the care of patients.”*
*“Since we care for our dialysis population as a group (patients are not attributed to an individual nephrologist), it is important to have buy-in from the entire group. Otherwise patients who start at 3 times per week, may routinely be increased to 3 times per week by a dissenting colleague during their rotation. It is not that I think it is not feasible. I believe this should be a question of individualized care. ID can be considered for all patients, but I would not be comfortable with a protocolized approach. I would like to mention that I use an incremental approach more widely in PD, but tend to start patients earlier. Also, in my experience, a step down approach, where patients start at 3 times per week and are stepped down if appropriate results in better adherence. This might not be the case if we had a formalized new patient education program.”*
*“Just want to highlight that for incremental dialysis to be feasible, there needs to be structured follow-up and continuity with a forum to routinely address whether goals of care are being met (ie. serial assessments of adequacy, residual renal function, complications of ESKD, and other comorbid conditions). It would not be appropriate if we cannot ensure continuity and a personalized approach.”*
*“Mentality of the unit and the ‘culture’. Staff usually very resistant to change. Will be upset with ‘more’ patients to look after. Dosing of incremental HD and shared model with lack of agreement amongst MDs as to goals and management.”*
*“I doubt most clinicians would want to engage in the degree of shared decision making and counselling required to make this possible without standardized criteria and the resistance to increasing dialysis time by patients longitudinally.”*
*“Shared care model so despite rounding on same shift can be gaps where you do not see patient: should one person continue to follow the patient’s labs, discussions to avoid patient being lost to the system.”*

*Note.* HD = hemodialysis; QOL = quality of life; CHF = congestive heart failure; ESLD = end-stage liver disease; eGFR = estimated glomerular filtration rate; RCT = randomized control trial; IDEAL = initiating dialysis early and late study; PD = peritoneal dialysis; ESKD = end-stage kidney disease.

a Content displayed is unedited from original statements.

### Opinions on Dialysis Prescribing Patterns, Including Incremental Hemodialysis

**Figure 2.**
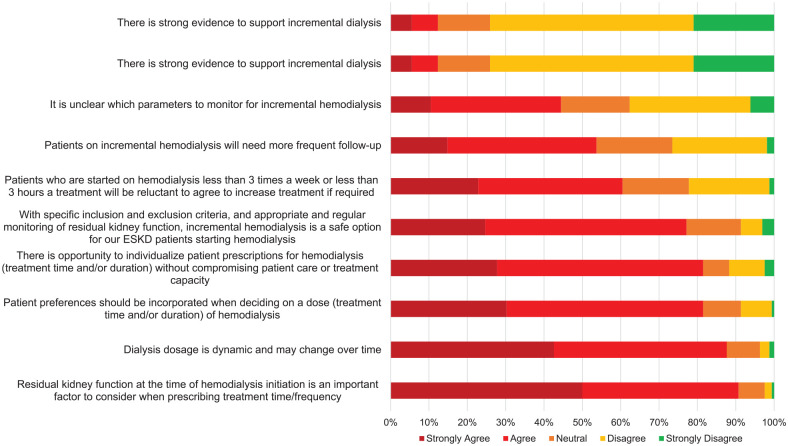
Degree of agreement with statements on dialysis prescriptions and incremental hemodialysis. *Note.* Responses are represented as a percentage of a total *n* = 162. ESKD = end-stage kidney disease.

Participants were asked to rank the degree of agreement with various statements on dialysis prescribing patterns, including incremental dialysis (Figure 2). Most of the participants agree that dialysis prescriptions are dynamic (88%) and RKF is an important component in prescribing hemodialysis (81%). Although 74% of participants disagreed that there is strong evidence supporting incremental dialysis, 78% agreed that with specific inclusion and exclusion criteria, incremental dialysis is a safe option. Participants who provided shift-based dialysis care were more likely to agree that with specific criteria, incremental hemodialysis can be a safe dialysis modality (*P* = .05) (results not shown).

### Barriers to Incremental Hemodialysis

**Figure 3.**
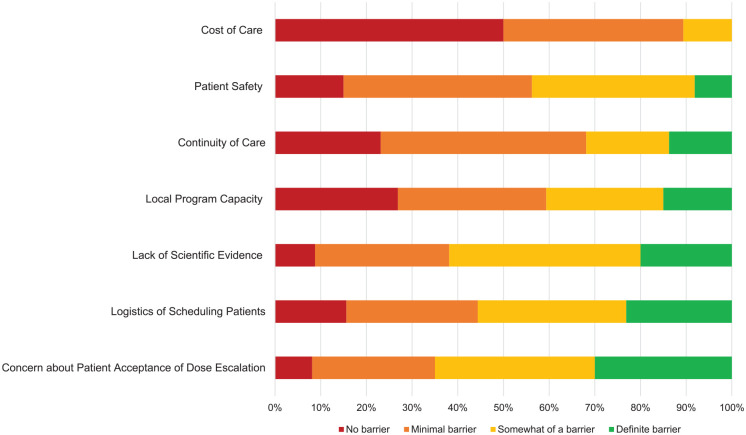
Potential barriers to incremental hemodialysis. *Note.* Responses are represented as a percentage of a total *n* = 160.

Participants were asked to rank a prespecified list of potential barriers to designing an incremental dialysis program (see Figure 3). Lack of scientific evidence was identified as a barrier to incremental hemodialysis. Sixty-five percent of participants agreed that patient acceptance of dose escalation is somewhat/definite barrier. Participants who provided shift-based dialysis care were more likely to identify patient acceptance of dose escalation as a barrier to incremental hemodialysis (*P* = .003) (results not shown). Furthermore, participants who work in an academic practice were more likely to identify continuity of care as a barrier to incremental hemodialysis (*P* = .047). Participants who completed their nephrology training in Canada were more likely to identify patient safety as a barrier to incremental hemodialysis (*P* = .0006).

Participants were asked to provide free-text responses on potential barriers to incremental hemodialysis (see Table 3). Simple thematic analysis revealed concerns regarding limited scientific evidence in the form of randomized trials. Furthermore, participants had stated that centers are often over-capacity and it will be challenging to accommodate the individual schedules required for incremental hemodialysis. Finally, participants expressed concern regarding cooperation of patients and staff members to the implementation of an incremental hemodialysis program. Despite the identified barriers, 82% of participants agreed that incremental hemodialysis is feasible with their current resources (see Table 4). Chi-square analysis did not reveal differences in the perception of incremental hemodialysis feasibility based on the participant demographic characteristics (results not shown).

**Table 4.** Feasibility and Potential Number of Patients to Benefit From Incremental Dialysis With Current Resources.

Variable, No. (%) unless otherwise specified	*n*	%
Potential percentage of hemodialysis patients that could benefit from incremental dialysis *n* = 161		
0%	13	8
<10%	43	27
10-25%	70	43
25-50%	25	16
>50%	10	6
Incremental dialysis is feasible with current resources *n* = 162		
Yes	132	82
No	30	18

## Discussion

This national survey of Canadian nephrologists indicates that the practice of incremental hemodialysis is widespread, but discretionary, with no systematic approach to assessment and monitoring. We identified that the 3 most common criteria used in the prescription of incremental hemodialysis are residual urine output, electrolyte homeostasis, and goals of care. No significant association was found between reimbursement model and prescription of incremental hemodialysis. For prescribers with a model available for incremental hemodialysis, an individualized approach was most used. In contrast, it was uncommon to start hemodialysis with reduced hours 3 times weekly or with once-weekly treatment. Significant concerns included patient safety, logistics of scheduling, lack of scientific evidence, and patient acceptance of dose escalation. However, most of the surveyed nephrologists felt that incremental hemodialysis can be safely prescribed.

Incremental dialysis, where the frequency and duration of dialysis sessions are individualized to account for RKF, has been discussed as a possible strategy to address the various patient- and system-related issues associated with conventional hemodialysis.^16^ An opinion paper published in 2014 proposed 10 criteria for patient eligibility for incremental hemodialysis: RKF, manageable intradialytic weight gain, stable cardiovascular status, infrequent hospitalization, quality of life, small to normal body habitus, good nutritional status, and absence of hyperkalemia, hyperphosphatemia, and profound anemia.^17^ A systematic review identified 15 observational studies comparing outcomes in patients receiving incremental hemodialysis with those receiving standard thrice-weekly treatment and found similar survival between groups, with greater preservation of RKF in those receiving incremental hemodialysis, deferring a requirement for a full dialysis dose by a mean of 12.1 months (95% confidence interval = 9.8-14.3).^18^ In addition to preservation of RKF, incremental hemodialysis has been associated with shorter recovery time after dialysis treatment, similar or improved health-related quality of life, and fewer dialysis access-related complications.^18,19^ Although incremental hemodialysis has been endorsed by national and regional dialysis guidelines, little is published on its actual practice. In North America and Europe, approximately 4% of hemodialysis patients are on twice-weekly or less frequent hemodialysis.^20,21^ In non-Western countries, this number is considerable higher, up to 75% in areas like Sudan, which may largely be driven by resource limitations.^22^ However, it should be noted that less frequent dialysis is not the same as incremental dialysis and it is not well known how many of these centers account for RKF when prescribing less frequent hemodialysis. Individual dialysis center experiences who have prescribed less frequent dialysis with monitoring of RKF have shown younger, less comorbid patients with Kt/V_urea_ of at least 1.6 tend to have similar survival outcomes as those prescribed thrice-weekly dialysis.^23-25^ Anecdotally, it is known that these centers have continued their practice of incremental dialysis, but there is limited information on how their programs have evolved over time. At this time, there is no clear consensus as to which patient and clinical factors should be taken into account when prescribing incremental dialysis. Randomized control trials currently in progress hope to address the efficacy of incremental dialysis.^26,27^

The results of this survey demonstrate broad agreement with previously identified clinical and patient factors, especially RKF, in prescribing incremental hemodialysis. Furthermore, we have shown there is a wide practice of incremental hemodialysis in Canada. For the first time, this survey has concretely identified barriers and concerns regarding the implementation of an incremental hemodialysis program. Development of clear eligibility and monitoring criteria, appropriate patient and provider education regarding treatment options, and a shared decision-making model in making dialysis treatment decisions should be considered in future development of incremental hemodialysis programs.

## Limitations

The generalizability of our study is limited by the response rate of 35%. We acknowledge there is a likely bias and potential overestimation of frequency of the provision of incremental hemodialysis: participants who provide incremental dialysis may be more likely to complete a survey on the subject. However, this is a known issue with any elecAtronic survey and our response rate was comparable with the usual rates seen in electronic surveys. Many survey participants were from an academic setting, making the results less generalizable to the community setting.

## Conclusions

Anecdotally, the use of incremental hemodialysis is known; however, there is a paucity of information on nephrologist opinions regarding and current practice of incremental hemodialysis. The results of this survey indicate that although the practice of incremental dialysis is widespread, there is a lack of standardization, resulting in a large variation in care. Going forward, efforts to implement an incremental hemodialysis program will need to address concerns regarding patient expectations regarding treatment, patient safety, and dialysis scheduling logistics.

## Supplemental Material

sj-docx-1-cjk-10.1177_20543581211065255 – Supplemental material for Knowledge and Practice of Incremental Hemodialysis: A Survey of Canadian Nephrologists Click here for additional data file. Supplemental material, sj-docx-1-cjk-10.1177_20543581211065255 for Knowledge and Practice of Incremental Hemodialysis: A Survey of Canadian Nephrologists by Anita Dahiya, Aminu Bello, Stephanie Thompson, Kara Schick-Makaroff and Neesh Pannu in Canadian Journal of Kidney Health and Disease

sj-docx-2-cjk-10.1177_20543581211065255 – Supplemental material for Knowledge and Practice of Incremental Hemodialysis: A Survey of Canadian Nephrologists Click here for additional data file. Supplemental material, sj-docx-2-cjk-10.1177_20543581211065255 for Knowledge and Practice of Incremental Hemodialysis: A Survey of Canadian Nephrologists by Anita Dahiya, Aminu Bello, Stephanie Thompson, Kara Schick-Makaroff and Neesh Pannu in Canadian Journal of Kidney Health and Disease
