# Supplementary material for: Knowledge and Practice of Incremental Hemodialysis: A Survey of Canadian Nephrologists
Source: Can J Kidney Health Dis. 2021 Dec 17;8:20543581211065255. doi: 10.1177/20543581211065255 (PMC8689607; doi:10.1177/20543581211065255)
Supplement: sj-docx-1-cjk-10.1177_20543581211065255 – Supplemental material for Knowledge and Practice of Incremental Hemodialysis: A Survey of Canadian Nephrologists [file sj-docx-1-cjk-10.1177_20543581211065255.docx]

**CJKHD-21-0079.R2**

**Title: Knowledge and practice of incremental hemodialysis: A survey of Canadian nephrologists**

**Original word count: 425**

**Contexte :** L’hémodialyse incrémentale est une stratégie tenant compte de la fonction rénale résiduelle pour individualiser la prescription de dialyse. Comparée à l’hémodialyse conventionnelle, cette modalité pourrait être associée à une amélioration de la qualité de vie des patients et à une réduction des coûts de santé.

**Objectif :** Nous avons interrogé des néphrologues canadiens en exercice afin d’évaluer leurs connaissances, leurs perceptions et leurs habitudes de pratique relativement à l’hémodialyse incrémentale.

**Conception de l’étude :** Une enquête transversale en ligne a été distribuée. Les questions portaient sur les pratiques de prescription de l’hémodialyse incrémentale, notamment sur la fréquence de prescription, les facteurs cliniques utilisés pour déterminer l’adéquation du traitement, et les obstacles à la mise en œuvre. Le sondage a été réalisé entre le 21 septembre et le 30 octobre 2020.

**Participants :** Nous avons distribué le sondage aux néphrologues canadiens en exercice figurant sur une liste privée des membres de la Société canadienne de néphrologie (SCN), ainsi qu’aux néphrologues figurant sur une liste nationale publique des néphrologues canadiens en exercice, créée à partir des registres des Collèges des médecins provinciaux. Il s’agit d’échantillons de commodité.

**Méthodologie :** Nous avons procédé à une analyse descriptive des données catégoriques, notamment des fréquences pour les variables nominales et des mesures de tendance centrale (moyenne) et de dispersion (écart-type) pour les variables ordinales. Nous avons utilisé l’analyse du chi-carré pour établir l’association des caractéristiques des répondants et de leurs pratiques avec leurs opinions et comportements à l’égard de la dialyse incrémentale. Nous avons procédé à une analyse thématique simple des réponses en texte libre aux questions portant sur la prescription de l’hémodialyse incrémentale, en se concentrant sur l’âge et la prise en charge initiale des comorbidités cardiaques et non cardiaques.

**Résultats :** Le taux de réponse était de 35 % (243/691). Une majorité de répondants (138/211 [65 %]) prescrivaient l’hémodialyse incrémentale selon une approche individualisée à la discrétion du néphrologue. La quasi-totalité des répondants (200/203 [98 %]) n’a pas fait état d’une politique de mise en œuvre. La diurèse résiduelle a été désignée comme le principal facteur d’admissibilité (112/172 [65 %]), suivie de la stabilité électrolytique (76/172 [44 %]) et des objectifs de soins du patient (69/117 [40 %]). La plupart des répondants ont convenu que la prescription de dialyse devrait tenir compte de la fonction rénale résiduelle; 74 % d’entre eux se sont toutefois dits en désaccord avec une affirmation selon laquelle il existe de solides preuves appuyant l’hémodialyse incrémentale. La sécurité du patient, l’acceptation par le patient d’une augmentation de la dose et la logistique de la planification figurent parmi les obstacles à la mise en œuvre mentionnés par les répondants. Malgré ces obstacles, 82 % des répondants étaient d’avis que l’hémodialyse incrémentale est possible avec les ressources actuelles, et 78 % étaient d’accord pour dire qu’avec des critères précis, cette modalité est une option sûre.

**Limites :** La généralisabilité de notre étude est limitée par son faible taux de réponse (35 %), bien que celui-ci soit comparable à ceux qu’on observe généralement pour les enquêtes électroniques. La plupart des répondants exerçaient dans un cadre académique, ce qui peut avoir introduit un biais dans les résultats.

**Conclusion :** L’hémodialyse incrémentale est fréquemment prescrite par les néphrologues canadiens, malgré une perception de preuves limitées et un manque d’orientation pour sa mise en œuvre. Les répondants ont mentionné quelques obstacles à sa mise en œuvre, soulignant ainsi la nécessité de mener des recherches afin de mieux orienter la pratique.
