# Supplementary material for: Knowledge and Practice of Incremental Hemodialysis: A Survey of Canadian Nephrologists
Source: Can J Kidney Health Dis. 2021 Dec 17;8:20543581211065255. doi: 10.1177/20543581211065255 (PMC8689607; doi:10.1177/20543581211065255)
Supplement: sj-docx-2-cjk-10.1177_20543581211065255 – Supplemental material for Knowledge and Practice of Incremental Hemodialysis: A Survey of Canadian Nephrologists [file sj-docx-2-cjk-10.1177_20543581211065255.docx]

| \| Incremental Dialysis - Canadian Nephrologists survey (August 2020)  [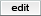](https://survey.albertahealthservices.ca/SurveyOptions.aspx?Mode=Edit&Nav=PageModifySurvey&SurveyID=llLLn86KL) \|  \|  \| \| --- \| --- \| --- \|  \|  \|  \| \| \|  \| Click on the "New Pg" button to add a new item on a new first page. \| [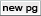](javascript:insertItemOnNewPage('',%20'1',%20'Insert',%20'0',%20'Yes');) \|  \| \| --- \| --- \| --- \| --- \| \|  \| \| \| [No Title Entered] \| \| --- \| \| \| --- \| --- \| \| [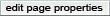](https://survey.albertahealthservices.ca/Page.aspx?SurveyID=llLLn86KL&PageNumber=1)[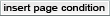](javascript:viewPageConditions('llLLn86KL',%20'1');)[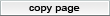](javascript:copyPage('llLLn86KL',%20'1');)[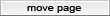](javascript:movePage('llLLn86KL',%20'1');)[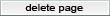](javascript:deletePage('llLLn86KL',%20'1');) \| \| \|  \| \|  \|  \| \|  \| \| \| --- \| --- \| --- \| --- \| --- \| --- \| --- \| --- \| --- \| --- \| --- \| --- \| --- \| --- \| --- \| --- \| \| \|  \|  \| \|  \| We are trying to determine current opinions and practices regarding incremental dialysis amongst Canadian nephrologists.  For the purposes of this survey incremental dialysis refers to prescription of dialysis in **new** Hemodialysis patients (within 12 months of starting treatment), where hemodialysis treatment is titrated according to the patient’s residual kidney function.    **"Incremental hemodialysis" refers to any treatment plan where the dialysis prescription at initiation is less than 3 times a week *or* less than 4 hours per treatment, where the intention is to increase treatment when necessary.  For the purposes of this study, we will focus on in-centre hemodialysis.** \| \| --- \| --- \| \|  \| \|  \| \| --- \| \| \|  \|  \| \| [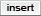](javascript:itemClicked('538365',%20'1',%20'Insert',%20'1');)[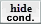](https://survey.albertahealthservices.ca/QuestionHideCondition.aspx?SurveyID=llLLn86KL&Item=538365)[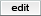](javascript:itemClicked('538365',%20'1',%20'Edit',%20'1');)[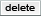](javascript:deleteItem('538365',%20'1',%20'llLLn86KL',%20'1');)[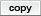](javascript:copyItem('538365',%20'1',%20'llLLn86KL',%20'1');)[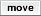](javascript:moveItem('538365',%20'1',%20'llLLn86KL',%20'1');)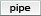 \|  \| \| --- \| --- \| --- \| --- \| --- \| --- \| --- \| --- \| --- \| --- \| --- \| --- \| \|  \|  \| \| \|  \| \|  \| Click on the "Insert" button to add an item here. Click on the "New Pg" button to add an item here on a new page. \| \| [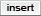](javascript:itemClicked('',%20'2',%20'Insert',%20'1');)[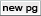](javascript:insertItemOnNewPage('',%20'1',%20'Insert',%20'1',%20'Yes');) \|  \| \| \|  \| \|  \| \| \|  \| \| \| Demographics \| \| --- \| \| \| --- \| --- \| \| [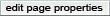](https://survey.albertahealthservices.ca/Page.aspx?SurveyID=llLLn86KL&PageNumber=2)[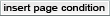](javascript:viewPageConditions('llLLn86KL',%20'2');)[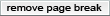](javascript:removePageBreak('llLLn86KL',%20'2');)[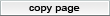](javascript:copyPage('llLLn86KL',%20'2');)[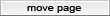](javascript:movePage('llLLn86KL',%20'2');)[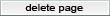](javascript:deletePage('llLLn86KL',%20'2');) \| \|  \| \| --- \| --- \| --- \| --- \| --- \| --- \| \|  \|  \|  \| \| \| \|  \| [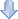](javascript:reorderItem('538362',%20'2',%20'Down');) \| \|  \| **Where did you complete your nephrology subspecialty training?*** \| \| --- \| --- \| \|  \| \|  \| Canada \| \| --- \| --- \| \|  \| United States \|  \| Other, please specify      \| \| --- \| \| \|  \|  \| \|  \|  \| \| [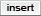](javascript:itemClicked('538362',%20'2',%20'Insert',%20'2');)[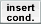](https://survey.albertahealthservices.ca/QuestionConditions.aspx?SurveyID=llLLn86KL&Item=538362)[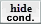](https://survey.albertahealthservices.ca/QuestionHideCondition.aspx?SurveyID=llLLn86KL&Item=538362)[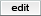](javascript:itemClicked('538362',%20'2',%20'Edit',%20'2');)[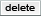](javascript:deleteItem('538362',%20'2',%20'llLLn86KL',%20'2');)[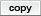](javascript:copyItem('538362',%20'2',%20'llLLn86KL',%20'2');)[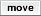](javascript:moveItem('538362',%20'2',%20'llLLn86KL',%20'2');)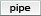 \|  \| \| --- \| --- \| --- \| --- \| --- \| --- \| --- \| --- \| --- \| --- \| --- \| --- \| --- \| --- \| --- \| --- \| --- \| --- \| \| \| \|  \|  \|  \| \| --- \| --- \| --- \| \| \| \|  \| [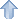](javascript:reorderItem('538363',%20'3',%20'Up');)[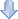](javascript:reorderItem('538363',%20'3',%20'Down');) \| \|  \| **How many years have you been in clinical practice?*** \| \| --- \| --- \| \|  \| \|  \| <5 years \| \| --- \| --- \| \|  \| 5 - 9 years \| \|  \| 10 - 19 years \| \|  \| > 20 years \|  \|  \| \| --- \| \| \|  \|  \| \|  \|  \| \| [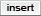](javascript:itemClicked('538363',%20'3',%20'Insert',%20'2');)[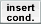](https://survey.albertahealthservices.ca/QuestionConditions.aspx?SurveyID=llLLn86KL&Item=538363)[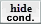](https://survey.albertahealthservices.ca/QuestionHideCondition.aspx?SurveyID=llLLn86KL&Item=538363)[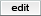](javascript:itemClicked('538363',%20'3',%20'Edit',%20'2');)[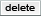](javascript:deleteItem('538363',%20'3',%20'llLLn86KL',%20'2');)[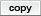](javascript:copyItem('538363',%20'3',%20'llLLn86KL',%20'2');)[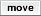](javascript:moveItem('538363',%20'3',%20'llLLn86KL',%20'2');)[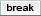](javascript:insertPageBreak('llLLn86KL',%20'3',%20'2');)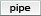 \|  \| \| --- \| --- \| --- \| --- \| --- \| --- \| --- \| --- \| --- \| --- \| --- \| --- \| --- \| --- \| --- \| --- \| --- \| --- \| --- \| --- \| --- \| --- \| \| \| \|  \|  \|  \| \| --- \| --- \| --- \| \| \| \|  \| [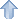](javascript:reorderItem('538364',%20'4',%20'Up');) \| \|  \| **In your practice, do you provide dialysis care for facility-based hemodialysis patients?*** \| \| --- \| --- \| \|  \| YesNo \| \|  \|  \| \| [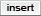](javascript:itemClicked('538364',%20'4',%20'Insert',%20'2');)[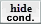](https://survey.albertahealthservices.ca/QuestionHideCondition.aspx?SurveyID=llLLn86KL&Item=538364)[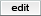](javascript:itemClicked('538364',%20'4',%20'Edit',%20'2');)[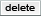](javascript:deleteItem('538364',%20'4',%20'llLLn86KL',%20'2');)[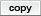](javascript:copyItem('538364',%20'4',%20'llLLn86KL',%20'2');)[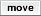](javascript:moveItem('538364',%20'4',%20'llLLn86KL',%20'2');)[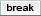](javascript:insertPageBreak('llLLn86KL',%20'4',%20'2');)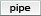 \|  \| \| --- \| --- \| --- \| --- \| --- \| --- \| --- \| --- \| --- \| --- \| --- \| \|  \|  \| \| \|  \| \|  \| Click on the "Insert" button to add an item here. Click on the "New Pg" button to add an item here on a new page. \| \| [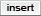](javascript:itemClicked('',%20'5',%20'Insert',%20'2');)[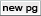](javascript:insertItemOnNewPage('',%20'4',%20'Insert',%20'2',%20'Yes');) \|  \| \| \|  \| \|  \| \| \|  \| \| \| Hemodialysis practice model \| \| --- \| \| \| --- \| --- \| \| [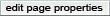](https://survey.albertahealthservices.ca/Page.aspx?SurveyID=llLLn86KL&PageNumber=3)[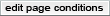](javascript:viewPageConditions('llLLn86KL',%20'3');)[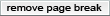](javascript:removePageBreak('llLLn86KL',%20'3');)[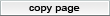](javascript:copyPage('llLLn86KL',%20'3');)[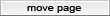](javascript:movePage('llLLn86KL',%20'3');)[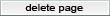](javascript:deletePage('llLLn86KL',%20'3');) \| \|  \| \| --- \| --- \| --- \| --- \| --- \| --- \| \|  \|  \|  \| \| \| \|  \| [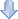](javascript:reorderItem('538344',%20'5',%20'Down');) \| \|  \| **How do physicians cover facility-based hemodialysis patients in your center (check all that apply)?*** \| \| --- \| --- \| \|  \| \|  \| We cover our own patients continuously \| \| --- \| --- \| \|  \| We cover dialysis shift(s) longitudinally throughout the year (intermittently or continuously) \| \|  \| We cover multiple shifts for short periods of time in blocks or rotations (not longitudinal) \| \|  \| Combination of the above (if so, please specify percentage of time at each level of care in the next question) \|   Other, please specify      \| \|  \|  \| \|  \|  \| \| [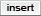](javascript:itemClicked('538344',%20'5',%20'Insert',%20'3');)[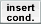](https://survey.albertahealthservices.ca/QuestionConditions.aspx?SurveyID=llLLn86KL&Item=538344)[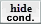](https://survey.albertahealthservices.ca/QuestionHideCondition.aspx?SurveyID=llLLn86KL&Item=538344)[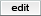](javascript:itemClicked('538344',%20'5',%20'Edit',%20'3');)[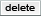](javascript:deleteItem('538344',%20'5',%20'llLLn86KL',%20'3');)[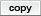](javascript:copyItem('538344',%20'5',%20'llLLn86KL',%20'3');)[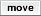](javascript:moveItem('538344',%20'5',%20'llLLn86KL',%20'3');)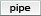 \|  \| \| --- \| --- \| --- \| --- \| --- \| --- \| --- \| --- \| --- \| --- \| --- \| --- \| --- \| --- \| --- \| --- \| --- \| --- \| --- \| --- \| --- \| \| \| \|  \|  \|  \| \| --- \| --- \| --- \| \| \| \|  \| [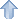](javascript:reorderItem('540434',%20'6',%20'Up');)[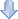](javascript:reorderItem('540434',%20'6',%20'Down');) \| \|  \| **In the question above, if you answered our physicians cover facility-based hemodialysis patients through a combination of levels of care, please indicate the percentage of time at each level of care.** \| \| --- \| --- \| \|  \| \| We cover dialysis shift(s) longitudinally throughout the year (intermittently or continuously) \|  \| \| --- \| --- \| \| We cover multiple shifts for short periods of time in blocks or rotations (not longitudinal) \|  \| \| \|  \|  \| \| [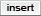](javascript:itemClicked('540434',%20'6',%20'Insert',%20'3');)[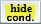](https://survey.albertahealthservices.ca/QuestionHideCondition.aspx?SurveyID=llLLn86KL&Item=540434)[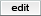](javascript:itemClicked('540434',%20'6',%20'Edit',%20'3');)[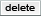](javascript:deleteItem('540434',%20'6',%20'llLLn86KL',%20'3');)[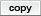](javascript:copyItem('540434',%20'6',%20'llLLn86KL',%20'3');)[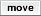](javascript:moveItem('540434',%20'6',%20'llLLn86KL',%20'3');)[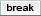](javascript:insertPageBreak('llLLn86KL',%20'6',%20'3');)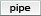 \|  \| \| --- \| --- \| --- \| --- \| --- \| --- \| --- \| --- \| --- \| --- \| --- \| --- \| --- \| --- \| --- \| \| \| \|  \|  \|  \| \| --- \| --- \| --- \| \| \| \|  \| [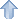](javascript:reorderItem('538345',%20'7',%20'Up');)[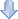](javascript:reorderItem('538345',%20'7',%20'Down');) \| \|  \| **If you cover your own patients continuously, do you see patients** \| \| --- \| --- \| \|  \| \|  \| Every treatment? \| \| --- \| --- \| \|  \| Weekly? \| \|  \| Bi-weekly? \| \|  \| Monthly \|  \| Other, please specify      \| \| --- \| \| \|  \|  \| \|  \|  \| \| [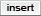](javascript:itemClicked('538345',%20'7',%20'Insert',%20'3');)[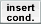](https://survey.albertahealthservices.ca/QuestionConditions.aspx?SurveyID=llLLn86KL&Item=538345)[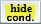](https://survey.albertahealthservices.ca/QuestionHideCondition.aspx?SurveyID=llLLn86KL&Item=538345)[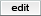](javascript:itemClicked('538345',%20'7',%20'Edit',%20'3');)[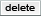](javascript:deleteItem('538345',%20'7',%20'llLLn86KL',%20'3');)[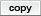](javascript:copyItem('538345',%20'7',%20'llLLn86KL',%20'3');)[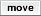](javascript:moveItem('538345',%20'7',%20'llLLn86KL',%20'3');)[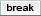](javascript:insertPageBreak('llLLn86KL',%20'7',%20'3');)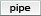 \|  \| \| --- \| --- \| --- \| --- \| --- \| --- \| --- \| --- \| --- \| --- \| --- \| --- \| --- \| --- \| --- \| --- \| --- \| --- \| --- \| --- \| --- \| --- \| \| \| \|  \|  \|  \| \| --- \| --- \| --- \| \| \| \|  \| [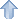](javascript:reorderItem('538346',%20'8',%20'Up');)[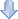](javascript:reorderItem('538346',%20'8',%20'Down');) \| \|  \| **If you cover dialysis shifts longitudinally throughout the year, do you care for hemodialysis patients** \| \| --- \| --- \| \|  \| \|  \| < 3 months per year \| \| --- \| --- \| \|  \| 4 - 6 months per year \| \|  \| > 6 months per year \|  \| Other, please specify      \| \| --- \| \| \|  \|  \| \|  \|  \| \| [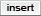](javascript:itemClicked('538346',%20'8',%20'Insert',%20'3');)[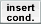](https://survey.albertahealthservices.ca/QuestionConditions.aspx?SurveyID=llLLn86KL&Item=538346)[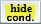](https://survey.albertahealthservices.ca/QuestionHideCondition.aspx?SurveyID=llLLn86KL&Item=538346)[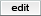](javascript:itemClicked('538346',%20'8',%20'Edit',%20'3');)[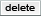](javascript:deleteItem('538346',%20'8',%20'llLLn86KL',%20'3');)[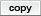](javascript:copyItem('538346',%20'8',%20'llLLn86KL',%20'3');)[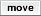](javascript:moveItem('538346',%20'8',%20'llLLn86KL',%20'3');)[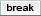](javascript:insertPageBreak('llLLn86KL',%20'8',%20'3');)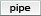 \|  \| \| --- \| --- \| --- \| --- \| --- \| --- \| --- \| --- \| --- \| --- \| --- \| --- \| --- \| --- \| --- \| --- \| --- \| --- \| --- \| --- \| \| \| \|  \|  \|  \| \| --- \| --- \| --- \| \| \| \|  \|  \| \|  \| **If you cover dialysis shifts longitudinally, do you see your patients (approximately):** \| \| --- \| --- \| \|  \| \|  \| Thrice weekly \| \| --- \| --- \| \|  \| Twice a week \| \|  \| Once a week \| \|  \| Once every 2 weeks \| \|  \| Once a month \|  \| Other, please specify \| \| --- \| \| \|  \|  \| \|  \|  \| \|  \|  \| \| --- \| --- \| --- \| --- \| --- \| --- \| --- \| --- \| --- \| --- \| --- \| --- \| --- \| --- \| --- \| --- \| --- \| --- \| --- \| --- \| --- \| --- \| --- \| --- \| \| \| \|  \|  \|  \| \| --- \| --- \| --- \| \| \| \|  \|  \| \|  \| **If you cover multiple shifts for short periods of time, do you care for hemodialysis patients** \| \| --- \| --- \| \|  \| \|  \| < 3 months per year \| \| --- \| --- \| \|  \| 4 - 6 months per year \| \|  \| > 6 months per year \|  \| Other, please specify \| \| --- \| \| \|  \|  \| \|  \|  \| \|  \|  \| \| --- \| --- \| --- \| --- \| --- \| --- \| --- \| --- \| --- \| --- \| --- \| --- \| --- \| --- \| --- \| --- \| --- \| --- \| --- \| --- \| \| \| \|  \|  \|  \| \| --- \| --- \| --- \| \| \| \|  \|  \| \|  \| **If you cover dialysis shifts multiple shifts for short periods of time, do you see your patients (approximately):** \| \| --- \| --- \| \|  \| \|  \| Thrice weekly \| \| --- \| --- \| \|  \| Twice a week \| \|  \| Once a week \| \|  \| Once every 2 weeks \| \|  \| Once a month \|  \| Other, please specify \| \| --- \| \| \|  \|  \| \|  \|  \| \|  \|  \| \| --- \| --- \| --- \| --- \| --- \| --- \| --- \| --- \| --- \| --- \| --- \| --- \| --- \| --- \| --- \| --- \| --- \| --- \| --- \| --- \| --- \| --- \| --- \| --- \| \|  \|  \| \| \|  \| \|  \| Click on the "Insert" button to add an item here. Click on the "New Pg" button to add an item here on a new page. \| \|  \|  \| \| \|  \| \|  \| \| \|  \| \| \| Practice environment and fee structure \| \| --- \| \| \| --- \| --- \| \|  \| \|  \| \| --- \| --- \| --- \| --- \| --- \| --- \| \|  \|  \|  \| \| \| \|  \|  \| \|  \| **What type of practice do you have?*** \| \| --- \| --- \| \|  \| \|  \| I work exclusively in an academic center \| \| --- \| --- \| \|  \| I have my own private practice (office outside academic center) \| \|  \| Mixed private and academic \|  \| Other, please specify \| \| --- \| \| \|  \|  \| \|  \|  \| \|  \|  \| \| --- \| --- \| --- \| --- \| --- \| --- \| --- \| --- \| --- \| --- \| --- \| --- \| --- \| --- \| --- \| --- \| --- \| --- \| --- \| --- \| \| \| \|  \|  \|  \| \| --- \| --- \| --- \| \| \| \|  \|  \| \|  \| **What proportion of your time do you spend doing clinical work?*** \| \| --- \| --- \| \|  \| \|  \| < 30 percent \| \| --- \| --- \| \|  \| 30 - 50 percent \| \|  \| 51 - 75 percent \| \|  \| > 75 percent \|  \|  \| \| --- \| \| \|  \|  \| \|  \|  \| \|  \|  \| \| --- \| --- \| --- \| --- \| --- \| --- \| --- \| --- \| --- \| --- \| --- \| --- \| --- \| --- \| --- \| --- \| --- \| --- \| --- \| --- \| --- \| --- \| \| \| \|  \|  \|  \| \| --- \| --- \| --- \| \| \| \|  \|  \| \|  \| **How are you reimbursed for dialysis coverage?*** \| \| --- \| --- \| \|  \| \|  \| Fee for service \| \| --- \| --- \| \|  \| Alternative relationship plan (fixed payment) \| \|  \| Prefer not to disclose \|  \| Other, please specify \| \| --- \| \| \|  \|  \| \|  \|  \| \|  \|  \| \| --- \| --- \| --- \| --- \| --- \| --- \| --- \| --- \| --- \| --- \| --- \| --- \| --- \| --- \| --- \| --- \| --- \| --- \| --- \| --- \| \|  \|  \| \| \|  \| \|  \| Click on the "Insert" button to add an item here. Click on the "New Pg" button to add an item here on a new page. \| \|  \|  \| \| \|  \| \|  \| \| \|  \| \| \| Incremental dialysis experience \| \| --- \| \| \| --- \| --- \| \|  \| \|  \| \| --- \| --- \| --- \| --- \| --- \| --- \| \|  \|  \|  \| \| \| \|  \|  \| \|  \| **Have you ever prescribed incremental hemodialysis?*** \| \| --- \| --- \| \|  \| YesNo \| \|  \|  \| \|  \|  \| \| --- \| --- \| --- \| --- \| --- \| --- \| --- \| --- \| --- \| --- \| --- \| \| \| \|  \|  \|  \| \| --- \| --- \| --- \| \| \| \|  \|  \| \|  \| **If you answered YES, please describe your current model.** \| \| --- \| --- \| \|  \|  \| \|  \|  \| \|  \|  \| \| --- \| --- \| --- \| --- \| --- \| --- \| --- \| --- \| --- \| --- \| --- \| \| \| \|  \|  \|  \| \| --- \| --- \| --- \| \| \| \|  \|  \| \|  \| **Does your center have specific criteria and/or a structured assessment program specifically for incremental hemodialysis**? \| \| --- \| --- \| \|  \| YesNo \| \|  \|  \| \|  \|  \| \| --- \| --- \| --- \| --- \| --- \| --- \| --- \| --- \| --- \| --- \| --- \| \| \| \|  \|  \|  \| \| --- \| --- \| --- \| \| \| \|  \|  \| \|  \| **How many in-centre hemodialysis patients are currently under your care?** \| \| --- \| --- \| \|  \| \|  \| 0 patients \| \| --- \| --- \| \|  \| < 10 patients \| \|  \| 10 to 50 patients \| \|  \| 51 - 100 patients \| \|  \| > 100 patients \| \|  \| unsure \|  \|  \| \| --- \| \| \|  \|  \| \|  \|  \| \|  \|  \| \| --- \| --- \| --- \| --- \| --- \| --- \| --- \| --- \| --- \| --- \| --- \| --- \| --- \| --- \| --- \| --- \| --- \| --- \| --- \| --- \| --- \| --- \| --- \| --- \| --- \| --- \| \| \| \|  \|  \|  \| \| --- \| --- \| --- \| \| \| \|  \|  \| \|  \| Of your in-centre hemodialysis patients, what percentage of new hemodialysis starts receive incremental dialysis? \| \| --- \| --- \| \|  \| \|  \| <30 percent \| \| --- \| --- \| \|  \| 30 - 50 percent \| \|  \| 51 - 75 percent \| \|  \| > 75 percent \| \|  \| unsure \|  \|  \| \| --- \| \| \|  \|  \| \|  \|  \| \|  \|  \| \| --- \| --- \| --- \| --- \| --- \| --- \| --- \| --- \| --- \| --- \| --- \| --- \| --- \| --- \| --- \| --- \| --- \| --- \| --- \| --- \| --- \| --- \| --- \| --- \| \|  \|  \| \| \|  \| \|  \| Click on the "Insert" button to add an item here. Click on the "New Pg" button to add an item here on a new page. \| \|  \|  \| \| \|  \| \|  \| \| \|  \| \| \| Incremental hemodialysis prescribing factors \| \| --- \| \| \| --- \| --- \| \|  \| \|  \| \| --- \| --- \| --- \| --- \| --- \| --- \| \|  \|  \|  \| \| \| \|  \|  \| \|  \| **Of the following options, which factors would you consider to be important the decision to initiate a patient on incremental hemodialysis?*** \| \| --- \| --- \| \|  \| \|  \|  \| **Very unimportant** \|  \| **Unimportant** \|  \| **Neutral** \|  \| **Important** \|  \| **Very Important** \|  \| **Critical** \|  \|  \| \| --- \| --- \| --- \| --- \| --- \| --- \| --- \| --- \| --- \| --- \| --- \| --- \| --- \| --- \| --- \| \| Transplantation status \|  \|  \|  \|  \|  \|  \|  \|  \|  \|  \|  \|  \|  \|  \| \| Employment status \|  \|  \|  \|  \|  \|  \|  \|  \|  \|  \|  \|  \|  \|  \| \| Distance of residence from dialysis unit \|  \|  \|  \|  \|  \|  \|  \|  \|  \|  \|  \|  \|  \|  \| \| Self-reported Quality of Life \|  \|  \|  \|  \|  \|  \|  \|  \|  \|  \|  \|  \|  \|  \| \| Electrolyte disturbances \|  \|  \|  \|  \|  \|  \|  \|  \|  \|  \|  \|  \|  \|  \| \| Blood pressure \|  \|  \|  \|  \|  \|  \|  \|  \|  \|  \|  \|  \|  \|  \| \| Urine output \|  \|  \|  \|  \|  \|  \|  \|  \|  \|  \|  \|  \|  \|  \| \| Patient's choice \|  \|  \|  \|  \|  \|  \|  \|  \|  \|  \|  \|  \|  \|  \| \| Patient adherence to follow up \|  \|  \|  \|  \|  \|  \|  \|  \|  \|  \|  \|  \|  \|  \| \| Body mass \|  \|  \|  \|  \|  \|  \|  \|  \|  \|  \|  \|  \|  \|  \| \| Goals of care \|  \|  \|  \|  \|  \|  \|  \|  \|  \|  \|  \|  \|  \|  \| \| Baseline management of cardiac co-morbidities (e.g., CHF, CAD, HTN, etc.) \|  \|  \|  \|  \|  \|  \|  \|  \|  \|  \|  \|  \|  \|  \| \| \|  \|  \| \|  \|  \| \|  \|  \| \| --- \| --- \| --- \| --- \| --- \| --- \| --- \| --- \| --- \| --- \| --- \| --- \| --- \| --- \| --- \| --- \| --- \| --- \| --- \| --- \| --- \| --- \| --- \| --- \| --- \| --- \| --- \| --- \| --- \| --- \| --- \| --- \| --- \| --- \| --- \| --- \| --- \| --- \| --- \| --- \| --- \| --- \| --- \| --- \| --- \| --- \| --- \| --- \| --- \| --- \| --- \| --- \| --- \| --- \| --- \| --- \| --- \| --- \| --- \| --- \| --- \| --- \| --- \| --- \| --- \| --- \| --- \| --- \| --- \| --- \| --- \| --- \| --- \| --- \| --- \| --- \| --- \| --- \| --- \| --- \| --- \| --- \| --- \| --- \| --- \| --- \| --- \| --- \| --- \| --- \| --- \| --- \| --- \| --- \| --- \| --- \| --- \| --- \| --- \| --- \| --- \| --- \| --- \| --- \| --- \| --- \| --- \| --- \| --- \| --- \| --- \| --- \| --- \| --- \| --- \| --- \| --- \| --- \| --- \| --- \| --- \| --- \| --- \| --- \| --- \| --- \| --- \| --- \| --- \| --- \| --- \| --- \| --- \| --- \| --- \| --- \| --- \| --- \| --- \| --- \| --- \| --- \| --- \| --- \| --- \| --- \| --- \| --- \| --- \| --- \| --- \| --- \| --- \| --- \| --- \| --- \| --- \| --- \| --- \| --- \| --- \| --- \| --- \| --- \| --- \| --- \| --- \| --- \| --- \| --- \| --- \| --- \| --- \| --- \| --- \| --- \| --- \| --- \| --- \| --- \| --- \| --- \| --- \| --- \| --- \| --- \| --- \| --- \| --- \| --- \| --- \| --- \| --- \| --- \| --- \| --- \| --- \| --- \| --- \| --- \| --- \| --- \| --- \| --- \| --- \| --- \| --- \| --- \| \| \| \|  \|  \|  \| \| --- \| --- \| --- \| \| \| \|  \|  \| \|  \| **Of the following options, which factors would you consider to be important the decision to initiate a patient on incremental hemodialysis?*** \| \| --- \| --- \| \|  \| \|  \|  \| **Very unimportant** \|  \| **Unimportant** \|  \| **Neutral** \|  \| **Important** \|  \| **Very Important** \|  \| **Critical** \|  \|  \| \| --- \| --- \| --- \| --- \| --- \| --- \| --- \| --- \| --- \| --- \| --- \| --- \| --- \| --- \| --- \| \| Age \|  \|  \|  \|  \|  \|  \|  \|  \|  \|  \|  \|  \|  \|  \| \| Etiology of renal failure \|  \|  \|  \|  \|  \|  \|  \|  \|  \|  \|  \|  \|  \|  \| \| Baseline management of non-cardiac related medical co-morbidities \|  \|  \|  \|  \|  \|  \|  \|  \|  \|  \|  \|  \|  \|  \| \| \|  \|  \| \|  \|  \| \|  \|  \| \| --- \| --- \| --- \| --- \| --- \| --- \| --- \| --- \| --- \| --- \| --- \| --- \| --- \| --- \| --- \| --- \| --- \| --- \| --- \| --- \| --- \| --- \| --- \| --- \| --- \| --- \| --- \| --- \| --- \| --- \| --- \| --- \| --- \| --- \| --- \| --- \| --- \| --- \| --- \| --- \| --- \| --- \| --- \| --- \| --- \| --- \| --- \| --- \| --- \| --- \| --- \| --- \| --- \| --- \| --- \| --- \| --- \| --- \| --- \| --- \| --- \| --- \| --- \| --- \| --- \| --- \| --- \| --- \| --- \| --- \| --- \| --- \| --- \| \| \| \|  \|  \|  \| \| --- \| --- \| --- \| \| \| \|  \|  \| \|  \| **If you answered AGE is important please indicate what about age factors in your decision.** \| \| --- \| --- \| \|  \|  \| \|  \|  \| \|  \|  \| \| --- \| --- \| --- \| --- \| --- \| --- \| --- \| --- \| --- \| --- \| --- \| \| \| \|  \|  \|  \| \| --- \| --- \| --- \| \| \| \|  \|  \| \|  \| **If you answered AGE is very important please indicate what about age factors in your decision.** \| \| --- \| --- \| \|  \|  \| \|  \|  \| \|  \|  \| \| --- \| --- \| --- \| --- \| --- \| --- \| --- \| --- \| --- \| --- \| --- \| \| \| \|  \|  \|  \| \| --- \| --- \| --- \| \| \| \|  \|  \| \|  \| **If you answered AGE is critical please indicate what about age factors in your decision.** \| \| --- \| --- \| \|  \|  \| \|  \|  \| \|  \|  \| \| --- \| --- \| --- \| --- \| --- \| --- \| --- \| --- \| --- \| --- \| --- \| \| \| \|  \|  \|  \| \| --- \| --- \| --- \| \| \| \|  \|  \| \|  \| **If you answered ETIOLOGY OF RENAL FAILURE is important please indicate how etiology of underlying renal failure would factor into your decision** \| \| --- \| --- \| \|  \|  \| \|  \|  \| \|  \|  \| \| --- \| --- \| --- \| --- \| --- \| --- \| --- \| --- \| --- \| --- \| --- \| \| \| \|  \|  \|  \| \| --- \| --- \| --- \| \| \| \|  \|  \| \|  \| **If you answered ETIOLOGY OF RENAL FAILURE is very important please indicate how etiology of underlying renal failure would factor into your decision** \| \| --- \| --- \| \|  \|  \| \|  \|  \| \|  \|  \| \| --- \| --- \| --- \| --- \| --- \| --- \| --- \| --- \| --- \| --- \| --- \| \| \| \|  \|  \|  \| \| --- \| --- \| --- \| \| \| \|  \|  \| \|  \| **If you answered ETIOLOGY OF RENAL FAILURE is critical please indicate how etiology of underlying renal failure would factor into your decision** \| \| --- \| --- \| \|  \|  \| \|  \|  \| \|  \|  \| \| --- \| --- \| --- \| --- \| --- \| --- \| --- \| --- \| --- \| --- \| --- \| \| \| \|  \|  \|  \| \| --- \| --- \| --- \| \| \| \|  \|  \| \|  \| **If you answered BASELINE MANAGEMENT OF NON-CARDIAC RELATED MEDICAL CO-MORBIDITIES is important please indicate which medical co-morbidities would be important to consider and why** \| \| --- \| --- \| \|  \|  \| \|  \|  \| \|  \|  \| \| --- \| --- \| --- \| --- \| --- \| --- \| --- \| --- \| --- \| --- \| --- \| \| \| \|  \|  \|  \| \| --- \| --- \| --- \| \| \| \|  \|  \| \|  \| **If you answered BASELINE MANAGEMENT OF NON-CARDIAC RELATED MEDICAL CO-MORBIDITIES is very important please indicate which medical co-morbidities would be important to consider and why** \| \| --- \| --- \| \|  \|  \| \|  \|  \| \|  \|  \| \| --- \| --- \| --- \| --- \| --- \| --- \| --- \| --- \| --- \| --- \| --- \| \| \| \|  \|  \|  \| \| --- \| --- \| --- \| \| \| \|  \|  \| \|  \| **If you answered BASELINE MANAGEMENT OF NON-CARDIAC RELATED MEDICAL CO-MORBIDITIES is critical please indicate which medical co-morbidities would be important to consider and why** \| \| --- \| --- \| \|  \|  \| \|  \|  \| \|  \|  \| \| --- \| --- \| --- \| --- \| --- \| --- \| --- \| --- \| --- \| --- \| --- \| \| \| \|  \|  \|  \| \| --- \| --- \| --- \| \| \| \|  \|  \| \|  \| **Of the above factors, please rank the three (3) factors that influence your decision the most.** \| \| --- \| --- \| \|  \| \|  \|  \| **Rating** \|  \| \| --- \| --- \| --- \| --- \| \| Most Important Factor \|  \|  \|  \| \| 2nd Most Important Factor \|  \|  \|  \| \| 3rd Most Important Factor \|  \|  \|  \| \| \|  \|  \| \|  \|  \| \| --- \| --- \| --- \| --- \| --- \| --- \| --- \| --- \| --- \| --- \| --- \| --- \| --- \| --- \| --- \| --- \| --- \| --- \| --- \| --- \| --- \| --- \| --- \| --- \| --- \| --- \| --- \| \| \| \|  \|  \|  \| \| --- \| --- \| --- \| \| \| \|  \|  \| \|  \| **What other factors would you consider?** \| \| --- \| --- \| \|  \|  \| \|  \|  \| \|  \|  \| \| --- \| --- \| --- \| --- \| --- \| --- \| --- \| --- \| --- \| --- \| --- \| \|  \|  \| \| \|  \| \|  \| Click on the "Insert" button to add an item here. Click on the "New Pg" button to add an item here on a new page. \| \|  \|  \| \| \|  \| \|  \| \| \|  \| \| \| [No Title Entered] \| \| --- \| \| \| --- \| --- \| \|  \| \|  \| \| --- \| --- \| --- \| --- \| --- \| --- \| \|  \|  \|  \| \| \| \|  \|  \| \|  \| **Reading the following statements please indicate your level of agreement:** \| \| --- \| --- \| \|  \| \|  \|  \| **Strongly Disagree** \|  \| **Disagree** \|  \| **Neutral** \|  \| **Agree** \|  \| **Strongly Agree** \|  \|  \| \| --- \| --- \| --- \| --- \| --- \| --- \| --- \| --- \| --- \| --- \| --- \| --- \| --- \| \| It is unclear which parameters to monitor for incremental hemodialysis \|  \|  \|  \|  \|  \|  \|  \|  \|  \|  \|  \|  \| \| Dialysis dosage is dynamic and may change over time \|  \|  \|  \|  \|  \|  \|  \|  \|  \|  \|  \|  \| \| Every patient starting hemodialysis should receive a minimal prescription of 4 hours three times a week \|  \|  \|  \|  \|  \|  \|  \|  \|  \|  \|  \|  \| \| With specific inclusion and exclusion criteria, and appropriate and regular monitoring of residual kidney function, incremental hemodialysis is a safe option for our ESKD patients starting hemodialysis \|  \|  \|  \|  \|  \|  \|  \|  \|  \|  \|  \|  \| \| Incremental hemodialysis will discourage patients from choosing home therapies \|  \|  \|  \|  \|  \|  \|  \|  \|  \|  \|  \|  \| \| Residual kidney function at the time of hemodialysis initiation is an important factor to consider when prescribing treatment time/frequency \|  \|  \|  \|  \|  \|  \|  \|  \|  \|  \|  \|  \| \| Patients who are started on hemodialysis less than 3 times a week or less than 3 hours a treatment will be reluctant to agree to increase treatment if required \|  \|  \|  \|  \|  \|  \|  \|  \|  \|  \|  \|  \| \| There is opportunity to individualize patient prescriptions for hemodialysis (treatment time and/or duration) without compromising patient care or treatment capacity \|  \|  \|  \|  \|  \|  \|  \|  \|  \|  \|  \|  \| \| Patient preferences should be incorporated when deciding on a dose (treatment time and/or duration) of hemodialysis \|  \|  \|  \|  \|  \|  \|  \|  \|  \|  \|  \|  \| \| Patients on incremental hemodialysis will need more frequent follow-up \|  \|  \|  \|  \|  \|  \|  \|  \|  \|  \|  \|  \| \| \|  \|  \| \|  \|  \| \|  \|  \| \| --- \| --- \| --- \| --- \| --- \| --- \| --- \| --- \| --- \| --- \| --- \| --- \| --- \| --- \| --- \| --- \| --- \| --- \| --- \| --- \| --- \| --- \| --- \| --- \| --- \| --- \| --- \| --- \| --- \| --- \| --- \| --- \| --- \| --- \| --- \| --- \| --- \| --- \| --- \| --- \| --- \| --- \| --- \| --- \| --- \| --- \| --- \| --- \| --- \| --- \| --- \| --- \| --- \| --- \| --- \| --- \| --- \| --- \| --- \| --- \| --- \| --- \| --- \| --- \| --- \| --- \| --- \| --- \| --- \| --- \| --- \| --- \| --- \| --- \| --- \| --- \| --- \| --- \| --- \| --- \| --- \| --- \| --- \| --- \| --- \| --- \| --- \| --- \| --- \| --- \| --- \| --- \| --- \| --- \| --- \| --- \| --- \| --- \| --- \| --- \| --- \| --- \| --- \| --- \| --- \| --- \| --- \| --- \| --- \| --- \| --- \| --- \| --- \| --- \| --- \| --- \| --- \| --- \| --- \| --- \| --- \| --- \| --- \| --- \| --- \| --- \| --- \| --- \| --- \| --- \| --- \| --- \| --- \| --- \| --- \| --- \| --- \| --- \| --- \| --- \| --- \| --- \| --- \| --- \| --- \| --- \| --- \| --- \| --- \| --- \| --- \| --- \| --- \| --- \| --- \| --- \| \| \| \|  \|  \|  \| \| --- \| --- \| --- \| \| \| \|  \|  \| \|  \| **Thinking of the last 20 patients you started on dialysis, how many of your patients do you see potentially benefiting from incremental hemodialysis?** \| \| --- \| --- \| \|  \| \|  \| none \| \| --- \| --- \| \|  \| < 10 percent \| \|  \| 10 - 25 percent \| \|  \| 25 - 50 percent \| \|  \| > 50 percent \|  \|  \| \| --- \| \| \|  \|  \| \|  \|  \| \|  \|  \| \| --- \| --- \| --- \| --- \| --- \| --- \| --- \| --- \| --- \| --- \| --- \| --- \| --- \| --- \| --- \| --- \| --- \| --- \| --- \| --- \| --- \| --- \| --- \| --- \| \| \| \|  \|  \|  \| \| --- \| --- \| --- \| \| \| \|  \|  \| \|  \| **With the current resources (staffing, beds, etc.) at the hemodialysis center(s) you are a part of, do you think an incremental hemodialysis program is feasible?*** \| \| --- \| --- \| \|  \| YesNo \| \|  \|  \| \|  \|  \| \| --- \| --- \| --- \| --- \| --- \| --- \| --- \| --- \| --- \| --- \| --- \| \| \| \|  \|  \|  \| \| --- \| --- \| --- \| \| \| \|  \|  \| \|  \| **If you do not think an incremental hemodialysis program is feasible, please explain why.** \| \| --- \| --- \| \|  \|  \| \|  \|  \| \|  \|  \| \| --- \| --- \| --- \| --- \| --- \| --- \| --- \| --- \| --- \| --- \| --- \| \| \| \|  \|  \|  \| \| --- \| --- \| --- \| \| \| \|  \|  \| \|  \| **Of the following, which barriers would you identify in considering an incremental hemodialysis program at your site:** \| \| --- \| --- \| \|  \| \|  \|  \| **No Barrier** \|  \| **Minimal Barrier** \|  \| **Somewhat of a Barrier** \|  \| **Definite Barrier** \|  \|  \| \| --- \| --- \| --- \| --- \| --- \| --- \| --- \| --- \| --- \| --- \| --- \| \| Concern about Patient Acceptance of Dose Escalation \|  \|  \|  \|  \|  \|  \|  \|  \|  \|  \| \| Lack of Scientific Evidence \|  \|  \|  \|  \|  \|  \|  \|  \|  \|  \| \| Continuity of Care \|  \|  \|  \|  \|  \|  \|  \|  \|  \|  \| \| Local Program Capacity \|  \|  \|  \|  \|  \|  \|  \|  \|  \|  \| \| Logistics of Scheduling Patients \|  \|  \|  \|  \|  \|  \|  \|  \|  \|  \| \| Patient Safety \|  \|  \|  \|  \|  \|  \|  \|  \|  \|  \| \| Cost of Care \|  \|  \|  \|  \|  \|  \|  \|  \|  \|  \| \| \|  \|  \| \|  \|  \| \|  \|  \| \| --- \| --- \| --- \| --- \| --- \| --- \| --- \| --- \| --- \| --- \| --- \| --- \| --- \| --- \| --- \| --- \| --- \| --- \| --- \| --- \| --- \| --- \| --- \| --- \| --- \| --- \| --- \| --- \| --- \| --- \| --- \| --- \| --- \| --- \| --- \| --- \| --- \| --- \| --- \| --- \| --- \| --- \| --- \| --- \| --- \| --- \| --- \| --- \| --- \| --- \| --- \| --- \| --- \| --- \| --- \| --- \| --- \| --- \| --- \| --- \| --- \| --- \| --- \| --- \| --- \| --- \| --- \| --- \| --- \| --- \| --- \| --- \| --- \| --- \| --- \| --- \| --- \| --- \| --- \| --- \| --- \| --- \| --- \| --- \| --- \| --- \| --- \| --- \| --- \| --- \| --- \| --- \| --- \| --- \| --- \| --- \| --- \| --- \| --- \| --- \| --- \| \| \| \|  \|  \|  \| \| --- \| --- \| --- \| \| \| \|  \|  \| \|  \| **What other barriers would you consider?** \| \| --- \| --- \| \|  \|  \| \|  \|  \| \|  \|  \| \| --- \| --- \| --- \| --- \| --- \| --- \| --- \| --- \| --- \| --- \| --- \| \| \| \|  \|  \|  \| \| --- \| --- \| --- \| \| \| \|  \|  \| \|  \| **Thank you for taking this survey   If you have specific questions regarding the survey, please contact Dr. Neesh Pannu at** [**npannu@ualberta.ca**](mailto:npannu@ualberta.ca) \| \| --- \| --- \| \|  \| \|  \| \| --- \| \| \|  \|  \| \|  \| \| --- \| --- \| --- \| --- \| --- \| --- \| --- \| --- \| --- \| --- \| --- \| \| \| \| --- \| --- \| --- \| --- \| --- \| --- \| --- \| --- \| --- \| --- \| --- \| --- \| --- \| --- \| --- \| --- \| --- \| --- \| --- \| --- \| --- \| --- \| --- \| --- \| --- \| --- \| --- \| --- \| --- \| --- \| --- \| --- \| --- \| --- \| --- \| --- \| --- \| --- \| --- \| --- \| --- \| --- \| --- \| --- \| --- \| --- \| --- \| --- \| --- \| --- \| --- \| --- \| --- \| --- \| --- \| --- \| --- \| --- \| --- \| --- \| --- \| --- \| --- \| --- \| --- \| --- \| --- \| --- \| --- \| --- \| --- \| --- \| --- \| --- \| --- \| --- \| --- \| --- \| --- \| --- \| --- \| --- \| --- \| --- \| --- \| --- \| --- \| --- \| --- \| --- \| --- \| --- \| --- \| --- \| --- \| --- \| --- \| --- \| --- \| --- \| --- \| --- \| --- \| --- \| --- \| --- \| --- \| --- \| --- \| --- \| --- \| --- \| --- \| --- \| --- \| --- \| --- \| --- \| --- \| --- \| --- \| --- \| --- \| --- \| --- \| --- \| --- \| --- \| --- \| --- \| --- \| --- \| --- \| --- \| --- \| --- \| --- \| --- \| --- \| --- \| --- \| --- \| --- \| --- \| --- \| --- \| --- \| --- \| --- \| --- \| --- \| --- \| --- \| --- \| --- \| --- \| --- \| --- \| --- \| --- \| --- \| --- \| --- \| --- \| --- \| --- \| --- \| --- \| --- \| --- \| --- \| --- \| --- \| --- \| --- \| --- \| --- \| --- \| --- \| --- \| --- \| --- \| --- \| --- \| --- \| --- \| --- \| --- \| --- \| --- \| --- \| --- \| --- \| --- \| --- \| --- \| --- \| --- \| --- \| --- \| --- \| --- \| --- \| --- \| --- \| --- \| --- \| --- \| --- \| --- \| --- \| --- \| --- \| --- \| --- \| --- \| --- \| --- \| --- \| --- \| --- \| --- \| --- \| --- \| --- \| --- \| --- \| --- \| --- \| --- \| --- \| --- \| --- \| --- \| --- \| --- \| --- \| --- \| --- \| --- \| --- \| --- \| --- \| --- \| --- \| --- \| --- \| --- \| --- \| --- \| --- \| --- \| --- \| --- \| --- \| --- \| --- \| --- \| --- \| --- \| --- \| --- \| --- \| --- \| --- \| --- \| --- \| --- \| --- \| --- \| --- \| --- \| --- \| --- \| --- \| --- \| --- \| --- \| --- \| --- \| --- \| --- \| --- \| --- \| --- \| --- \| --- \| --- \| --- \| --- \| --- \| --- \| --- \| --- \| --- \| --- \| --- \| --- \| --- \| --- \| --- \| --- \| --- \| --- \| --- \| --- \| --- \| --- \| --- \| --- \| --- \| --- \| --- \| --- \| --- \| --- \| --- \| --- \| --- \| --- \| --- \| --- \| --- \| --- \| --- \| --- \| --- \| --- \| --- \| --- \| --- \| --- \| --- \| --- \| --- \| --- \| --- \| --- \| --- \| --- \| --- \| --- \| --- \| --- \| --- \| --- \| --- \| --- \| --- \| --- \| --- \| --- \| --- \| --- \| --- \| --- \| --- \| --- \| --- \| --- \| --- \| --- \| --- \| --- \| --- \| --- \| --- \| --- \| --- \| --- \| --- \| --- \| --- \| --- \| --- \| --- \| --- \| --- \| --- \| --- \| --- \| --- \| --- \| --- \| --- \| --- \| --- \| --- \| --- \| --- \| --- \| --- \| --- \| --- \| --- \| --- \| --- \| --- \| --- \| --- \| --- \| --- \| --- \| --- \| --- \| --- \| --- \| --- \| --- \| --- \| --- \| --- \| --- \| --- \| --- \| --- \| --- \| --- \| --- \| --- \| --- \| --- \| --- \| --- \| --- \| --- \| --- \| --- \| --- \| --- \| --- \| --- \| --- \| --- \| --- \| --- \| --- \| --- \| --- \| --- \| --- \| --- \| --- \| --- \| --- \| --- \| --- \| --- \| --- \| --- \| --- \| --- \| --- \| --- \| --- \| --- \| --- \| --- \| --- \| --- \| --- \| --- \| --- \| --- \| --- \| --- \| --- \| --- \| --- \| --- \| --- \| --- \| --- \| --- \| --- \| --- \| --- \| --- \| --- \| --- \| --- \| --- \| --- \| --- \| --- \| --- \| --- \| --- \| --- \| --- \| --- \| --- \| --- \| --- \| --- \| --- \| --- \| --- \| --- \| --- \| --- \| --- \| --- \| --- \| --- \| --- \| --- \| --- \| --- \| --- \| --- \| --- \| --- \| --- \| --- \| --- \| --- \| --- \| --- \| --- \| --- \| --- \| --- \| --- \| --- \| --- \| --- \| --- \| --- \| --- \| --- \| --- \| --- \| --- \| --- \| --- \| --- \| --- \| --- \| --- \| --- \| --- \| --- \| --- \| --- \| --- \| --- \| --- \| --- \| --- \| --- \| --- \| --- \| --- \| --- \| --- \| --- \| --- \| --- \| --- \| --- \| --- \| --- \| --- \| --- \| --- \| --- \| --- \| --- \| --- \| --- \| --- \| --- \| --- \| --- \| --- \| --- \| --- \| --- \| --- \| --- \| --- \| --- \| --- \| --- \| --- \| --- \| --- \| --- \| --- \| --- \| --- \| --- \| --- \| --- \| --- \| --- \| --- \| --- \| --- \| --- \| --- \| --- \| --- \| --- \| --- \| --- \| --- \| --- \| --- \| --- \| --- \| --- \| --- \| --- \| --- \| --- \| --- \| --- \| --- \| --- \| --- \| --- \| --- \| --- \| --- \| --- \| --- \| --- \| --- \| --- \| --- \| --- \| --- \| --- \| --- \| --- \| --- \| --- \| --- \| --- \| --- \| --- \| --- \| --- \| --- \| --- \| --- \| --- \| --- \| --- \| --- \| --- \| --- \| --- \| --- \| --- \| --- \| --- \| --- \| --- \| --- \| --- \| --- \| --- \| --- \| --- \| --- \| --- \| --- \| --- \| --- \| --- \| --- \| --- \| --- \| --- \| --- \| --- \| --- \| --- \| --- \| --- \| --- \| --- \| --- \| --- \| --- \| --- \| --- \| --- \| --- \| --- \| --- \| --- \| --- \| --- \| --- \| --- \| --- \| --- \| --- \| --- \| --- \| --- \| --- \| --- \| --- \| --- \| --- \| --- \| --- \| --- \| --- \| --- \| --- \| --- \| --- \| --- \| --- \| --- \| --- \| --- \| --- \| --- \| --- \| --- \| --- \| --- \| --- \| --- \| --- \| --- \| --- \| --- \| --- \| --- \| --- \| --- \| --- \| --- \| --- \| --- \| --- \| --- \| --- \| --- \| --- \| --- \| --- \| --- \| --- \| --- \| --- \| --- \| --- \| --- \| --- \| --- \| --- \| --- \| --- \| --- \| --- \| --- \| --- \| --- \| --- \| --- \| --- \| --- \| --- \| --- \| --- \| --- \| --- \| --- \| --- \| --- \| --- \| --- \| --- \| --- \| --- \| --- \| --- \| --- \| --- \| --- \| --- \| --- \| --- \| --- \| --- \| --- \| --- \| --- \| --- \| --- \| --- \| --- \| --- \| --- \| --- \| --- \| --- \| --- \| --- \| --- \| --- \| --- \| --- \| --- \| --- \| --- \| --- \| --- \| --- \| --- \| --- \| --- \| --- \| --- \| --- \| --- \| --- \| --- \| --- \| --- \| --- \| --- \| --- \| --- \| --- \| --- \| --- \| --- \| --- \| --- \| --- \| --- \| --- \| --- \| --- \| --- \| --- \| --- \| --- \| --- \| --- \| --- \| --- \| --- \| --- \| --- \| --- \| --- \| --- \| --- \| --- \| --- \| --- \| --- \| --- \| --- \| --- \| --- \| --- \| --- \| --- \| --- \| --- \| --- \| --- \| --- \| --- \| --- \| --- \| --- \| --- \| --- \| --- \| --- \| --- \| --- \| --- \| --- \| --- \| --- \| --- \| --- \| --- \| --- \| --- \| --- \| --- \| --- \| --- \| --- \| --- \| --- \| --- \| --- \| --- \| --- \| --- \| --- \| --- \| --- \| --- \| --- \| --- \| --- \| --- \| --- \| --- \| --- \| --- \| --- \| --- \| --- \| --- \| --- \| --- \| --- \| --- \| --- \| --- \| --- \| --- \| --- \| --- \| --- \| --- \| --- \| --- \| --- \| --- \| --- \| --- \| --- \| --- \| --- \| --- \| --- \| --- \| --- \| --- \| --- \| --- \| --- \| --- \| --- \| --- \| --- \| --- \| --- \| --- \| --- \| --- \| --- \| --- \| --- \| --- \| --- \| --- \| --- \| --- \| --- \| --- \| --- \| --- \| --- \| --- \| --- \| --- \| --- \| --- \| --- \| --- \| --- \| --- \| --- \| --- \| --- \| --- \| --- \| --- \| --- \| --- \| --- \| --- \| --- \| --- \| --- \| --- \| --- \| --- \| --- \| --- \| --- \| --- \| --- \| --- \| --- \| --- \| --- \| --- \| --- \| --- \| --- \| --- \| --- \| --- \| --- \| --- \| --- \| --- \| --- \| --- \| --- \| --- \| --- \| --- \| --- \| --- \| --- \| --- \| --- \| --- \| --- \| --- \| --- \| --- \| --- \| --- \| --- \| --- \| --- \| --- \| --- \| --- \| --- \| --- \| --- \| --- \| --- \| --- \| --- \| --- \| --- \| --- \| --- \| --- \| --- \| --- \| --- \| --- \| --- \| --- \| --- \| --- \| --- \| --- \| --- \| --- \| --- \| --- \| --- \| --- \| --- \| --- \| --- \| --- \| --- \| --- \| --- \| --- \| --- \| --- \| --- \| --- \| --- \| --- \| --- \| --- \| --- \| --- \| --- \| --- \| --- \| --- \| --- \| --- \| --- \| --- \| --- \| --- \| --- \| --- \| --- \| --- \| --- \| --- \| --- \| --- \| --- \| --- \| --- \| --- \| --- \| --- \| --- \| --- \| --- \| --- \| --- \| --- \| --- \| --- \| --- \| --- \| --- \| --- \| --- \| --- \| --- \| --- \| --- \| --- \| --- \| --- \| --- \| --- \| --- \| --- \| --- \| --- \| --- \| --- \| --- \| --- \| --- \| --- \| --- \| --- \| --- \| --- \| --- \| --- \| --- \| --- \| --- \| --- \| --- \| --- \| --- \| --- \| --- \| --- \| --- \| --- \| --- \| --- \| --- \| --- \| --- \| --- \| --- \| --- \| --- \| --- \| --- \| --- \| --- \| --- \| --- \| --- \| --- \| --- \| --- \| --- \| --- \| --- \| --- \| --- \| --- \| --- \| --- \| --- \| --- \| --- \| --- \| --- \| --- \| --- \| --- \| --- \| --- \| --- \| --- \| --- \| --- \| --- \| --- \| --- \| --- \| --- \| --- \| --- \| --- \| --- \| --- \| --- \| --- \| --- \| --- \| --- \| --- \| --- \| --- \| --- \| --- \| --- \| --- \| --- \| --- \| --- \| --- \| --- \| --- \| --- \| --- \| --- \| --- \| --- \| --- \| --- \| --- \| --- \| --- \| --- \| --- \| --- \| --- \| --- \| --- \| --- \| --- \| --- \| --- \| --- \| --- \| --- \| --- \| --- \| --- \| --- \| --- \| --- \| --- \| --- \| --- \| --- \| --- \| --- \| --- \| --- \| --- \| --- \| --- \| --- \| --- \| --- \| --- \| --- \| --- \| --- \| --- \| --- \| --- \| --- \| --- \| --- \| --- \| --- \| --- \| --- \| --- \| --- \| --- \| --- \| --- \| --- \| --- \| --- \| --- \| --- \| --- \| --- \| --- \| --- \| --- \| --- \| --- \| --- \| --- \| --- \| --- \| --- \| --- \| --- \| --- \| --- \| --- \| --- \| --- \| --- \| --- \| --- \| --- \| --- \| --- \| --- \| --- \| --- \| --- \| --- \| --- \| --- \| --- \| --- \| --- \| --- \| --- \| --- \| --- \| --- \| --- \| --- \| --- \| --- \| --- \| --- \| --- \| --- \| --- \| --- \| --- \| --- \| --- \| --- \| --- \| --- \| --- \| --- \| --- \| --- \| --- \| --- \| --- \| --- \| --- \| --- \| --- \| --- \| --- \| --- \| --- \| --- \| --- \| --- \| --- \| --- \| --- \| --- \| --- \| --- \| --- \| --- \| --- \| --- \| --- \| --- \| --- \| --- \| --- \| --- \| --- \| --- \| --- \| --- \| --- \| --- \| --- \| --- \| --- \| --- \| --- \| --- \| --- \| --- \| --- \| --- \| --- \| --- \| --- \| --- \| --- \| --- \| --- \| --- \| --- \| --- \| --- \| |  |  |
| --- | --- | --- | --- | --- | --- | --- | --- | --- | --- | --- | --- | --- | --- | --- | --- | --- | --- | --- | --- | --- | --- | --- | --- | --- | --- | --- | --- | --- | --- | --- | --- | --- | --- | --- | --- | --- | --- | --- | --- | --- | --- | --- | --- | --- | --- | --- | --- | --- | --- | --- | --- | --- | --- | --- | --- | --- | --- | --- | --- | --- | --- | --- | --- | --- | --- | --- | --- | --- | --- | --- | --- | --- | --- | --- | --- | --- | --- | --- | --- | --- | --- | --- | --- | --- | --- | --- | --- | --- | --- | --- | --- | --- | --- | --- | --- | --- | --- | --- | --- | --- | --- | --- | --- | --- | --- | --- | --- | --- | --- | --- | --- | --- | --- | --- | --- | --- | --- | --- | --- | --- | --- | --- | --- | --- | --- | --- | --- | --- | --- | --- | --- | --- | --- | --- | --- | --- | --- | --- | --- | --- | --- | --- | --- | --- | --- | --- | --- | --- | --- | --- | --- | --- | --- | --- | --- | --- | --- | --- | --- | --- | --- | --- | --- | --- | --- | --- | --- | --- | --- | --- | --- | --- | --- | --- | --- | --- | --- | --- | --- | --- | --- | --- | --- | --- | --- | --- | --- | --- | --- | --- | --- | --- | --- | --- | --- | --- | --- | --- | --- | --- | --- | --- | --- | --- | --- | --- | --- | --- | --- | --- | --- | --- | --- | --- | --- | --- | --- | --- | --- | --- | --- | --- | --- | --- | --- | --- | --- | --- | --- | --- | --- | --- | --- | --- | --- | --- | --- | --- | --- | --- | --- | --- | --- | --- | --- | --- | --- | --- | --- | --- | --- | --- | --- | --- | --- | --- | --- | --- | --- | --- | --- | --- | --- | --- | --- | --- | --- | --- | --- | --- | --- | --- | --- | --- | --- | --- | --- | --- | --- | --- | --- | --- | --- | --- | --- | --- | --- | --- | --- | --- | --- | --- | --- | --- | --- | --- | --- | --- | --- | --- | --- | --- | --- | --- | --- | --- | --- | --- | --- | --- | --- | --- | --- | --- | --- | --- | --- | --- | --- | --- | --- | --- | --- | --- | --- | --- | --- | --- | --- | --- | --- | --- | --- | --- | --- | --- | --- | --- | --- | --- | --- | --- | --- | --- | --- | --- | --- | --- | --- | --- | --- | --- | --- | --- | --- | --- | --- | --- | --- | --- | --- | --- | --- | --- | --- | --- | --- | --- | --- | --- | --- | --- | --- | --- | --- | --- | --- | --- | --- | --- | --- | --- | --- | --- | --- | --- | --- | --- | --- | --- | --- | --- | --- | --- | --- | --- | --- | --- | --- | --- | --- | --- | --- | --- | --- | --- | --- | --- | --- | --- | --- | --- | --- | --- | --- | --- | --- | --- | --- | --- | --- | --- | --- | --- | --- | --- | --- | --- | --- | --- | --- | --- | --- | --- | --- | --- | --- | --- | --- | --- | --- | --- | --- | --- | --- | --- | --- | --- | --- | --- | --- | --- | --- | --- | --- | --- | --- | --- | --- | --- | --- | --- | --- | --- | --- | --- | --- | --- | --- | --- | --- | --- | --- | --- | --- | --- | --- | --- | --- | --- | --- | --- | --- | --- | --- | --- | --- | --- | --- | --- | --- | --- | --- | --- | --- | --- | --- | --- | --- | --- | --- | --- | --- | --- | --- | --- | --- | --- | --- | --- | --- | --- | --- | --- | --- | --- | --- | --- | --- | --- | --- | --- | --- | --- | --- | --- | --- | --- | --- | --- | --- | --- | --- | --- | --- | --- | --- | --- | --- | --- | --- | --- | --- | --- | --- | --- | --- | --- | --- | --- | --- | --- | --- | --- | --- | --- | --- | --- | --- | --- | --- | --- | --- | --- | --- | --- | --- | --- | --- | --- | --- | --- | --- | --- | --- | --- | --- | --- | --- | --- | --- | --- | --- | --- | --- | --- | --- | --- | --- | --- | --- | --- | --- | --- | --- | --- | --- | --- | --- | --- | --- | --- | --- | --- | --- | --- | --- | --- | --- | --- | --- | --- | --- | --- | --- | --- | --- | --- | --- | --- | --- | --- | --- | --- | --- | --- | --- | --- | --- | --- | --- | --- | --- | --- | --- | --- | --- | --- | --- | --- | --- | --- | --- | --- | --- | --- | --- | --- | --- | --- | --- | --- | --- | --- | --- | --- | --- | --- | --- | --- | --- | --- | --- | --- | --- | --- | --- | --- | --- | --- | --- | --- | --- | --- | --- | --- | --- | --- | --- | --- | --- | --- | --- | --- | --- | --- | --- | --- | --- | --- | --- | --- | --- | --- | --- | --- | --- | --- | --- | --- | --- | --- | --- | --- | --- | --- | --- | --- | --- | --- | --- | --- | --- | --- | --- | --- | --- | --- | --- | --- | --- | --- | --- | --- | --- | --- | --- | --- | --- | --- | --- | --- | --- | --- | --- | --- | --- | --- | --- | --- | --- | --- | --- | --- | --- | --- | --- | --- | --- | --- | --- | --- | --- | --- | --- | --- | --- | --- | --- | --- | --- | --- | --- | --- | --- | --- | --- | --- | --- | --- | --- | --- | --- | --- | --- | --- | --- | --- | --- | --- | --- | --- | --- | --- | --- | --- | --- | --- | --- | --- | --- | --- | --- | --- | --- | --- | --- | --- | --- | --- | --- | --- | --- | --- | --- | --- | --- | --- | --- | --- | --- | --- | --- | --- | --- | --- | --- | --- | --- | --- | --- | --- | --- | --- | --- | --- | --- | --- | --- | --- | --- | --- | --- | --- | --- | --- | --- | --- | --- | --- | --- | --- | --- | --- | --- | --- | --- | --- | --- | --- | --- | --- | --- | --- | --- | --- | --- | --- | --- | --- | --- | --- | --- | --- | --- | --- | --- | --- | --- | --- | --- | --- | --- | --- | --- | --- | --- | --- | --- | --- | --- | --- | --- | --- | --- | --- | --- | --- | --- | --- | --- | --- | --- | --- | --- | --- | --- | --- | --- | --- | --- | --- | --- | --- | --- | --- | --- | --- | --- | --- | --- | --- | --- | --- | --- | --- | --- | --- | --- | --- | --- | --- | --- | --- | --- | --- | --- | --- | --- | --- | --- | --- | --- | --- | --- | --- | --- | --- | --- | --- | --- | --- | --- | --- | --- | --- | --- | --- | --- | --- | --- | --- | --- | --- | --- | --- | --- | --- | --- | --- | --- | --- | --- | --- | --- | --- | --- | --- | --- | --- | --- | --- | --- | --- | --- | --- | --- | --- | --- | --- | --- | --- | --- | --- | --- | --- | --- | --- | --- | --- | --- | --- | --- | --- | --- | --- | --- | --- | --- | --- | --- | --- | --- | --- | --- | --- | --- | --- | --- | --- | --- | --- | --- | --- | --- | --- | --- | --- | --- | --- | --- | --- | --- | --- | --- | --- | --- | --- | --- | --- | --- | --- | --- | --- | --- | --- | --- | --- | --- | --- | --- | --- | --- | --- | --- | --- | --- | --- | --- | --- | --- | --- | --- | --- | --- | --- | --- | --- | --- | --- | --- | --- | --- | --- | --- | --- | --- | --- | --- | --- | --- | --- | --- | --- | --- | --- | --- | --- | --- | --- | --- | --- | --- | --- | --- | --- | --- | --- | --- | --- | --- | --- | --- | --- | --- | --- | --- | --- | --- | --- | --- | --- | --- | --- | --- | --- | --- | --- | --- | --- | --- | --- | --- | --- | --- | --- | --- | --- | --- | --- | --- | --- | --- | --- | --- | --- | --- | --- | --- | --- | --- | --- | --- | --- | --- | --- | --- | --- | --- | --- | --- | --- | --- | --- | --- | --- | --- | --- | --- | --- | --- | --- | --- | --- | --- | --- | --- | --- | --- | --- | --- | --- | --- | --- | --- | --- | --- | --- | --- | --- | --- | --- | --- | --- | --- | --- | --- | --- | --- | --- | --- | --- | --- | --- | --- | --- | --- | --- | --- | --- | --- | --- | --- | --- | --- | --- | --- | --- | --- | --- | --- | --- | --- | --- | --- | --- | --- | --- | --- | --- | --- | --- | --- | --- | --- | --- | --- | --- | --- | --- | --- | --- | --- | --- | --- | --- | --- | --- | --- | --- | --- | --- | --- | --- | --- | --- | --- | --- | --- | --- | --- | --- | --- | --- | --- | --- | --- | --- | --- | --- | --- | --- | --- | --- | --- | --- | --- | --- | --- | --- | --- | --- | --- | --- | --- | --- | --- | --- | --- | --- | --- | --- | --- | --- | --- | --- | --- | --- | --- | --- | --- | --- | --- | --- | --- | --- | --- | --- | --- | --- | --- | --- | --- | --- | --- | --- | --- | --- | --- | --- | --- | --- | --- | --- | --- | --- | --- | --- | --- | --- | --- | --- | --- | --- | --- | --- | --- | --- | --- | --- | --- | --- | --- | --- | --- | --- | --- | --- | --- | --- | --- | --- | --- | --- | --- | --- | --- | --- | --- | --- | --- | --- | --- | --- | --- | --- | --- | --- | --- | --- | --- | --- | --- | --- | --- | --- | --- | --- | --- | --- | --- | --- | --- | --- | --- | --- | --- | --- | --- | --- | --- | --- | --- | --- | --- | --- | --- | --- | --- | --- | --- | --- | --- | --- | --- | --- | --- | --- | --- | --- | --- | --- | --- | --- | --- | --- | --- | --- | --- | --- | --- | --- | --- | --- | --- | --- | --- | --- | --- | --- | --- | --- | --- | --- | --- | --- | --- | --- | --- | --- |
